# Deficiency of WTAP in hepatocytes induces lipoatrophy and non-alcoholic steatohepatitis (NASH)

**DOI:** 10.1038/s41467-022-32163-w

**Published:** 2022-08-04

**Authors:** Xinzhi Li, Kaixin Ding, Xueying Li, Bingchuan Yuan, Yuqin Wang, Zhicheng Yao, Shuaikang Wang, He Huang, Bolin Xu, Liwei Xie, Tuo Deng, Xiao-wei Chen, Zheng Chen

**Affiliations:** 1 HIT Center for Life Sciences, School of Life Science and Technology, Harbin Institute of Technology, Harbin, 150001 China; 2 Department of General Surgery, The Third Affiliated Hospital of Sun Yat-sen University, Guangzhou, China; 3 Shanghai Key Laboratory of Metabolic Remodeling and Health, Institute of Metabolism and Integrative Biology, Fudan University, Shanghai, 200438 China; 4 State Key Laboratory of Membrane Biology, Institute of Molecular Medicine, College of Future Technology, and Center for Life Sciences, Peking University, Beijing, 100871 China; 5 Guangdong Provincial Key Laboratory of Microbial Culture Collection and Application, State Key Laboratory of Applied Microbiology Southern China, Institute of Microbiology, Guangdong Academy of Sciences, Guangzhou, 510070 China; 6 National Clinical Research Center for Metabolic Diseases, Key Laboratory of Diabetes Immunology, Ministry of Education, and Department of Metabolism and Endocrinology, The Second Xiangya Hospital of Central South University, Changsha, 410011 Hunan China

**Keywords:** Non-alcoholic steatohepatitis, Dyslipidaemias

## Abstract

Ectopic lipid accumulation and inflammation are the essential signs of NASH. However, the molecular mechanisms of ectopic lipid accumulation and inflammation during NASH progression are not fully understood. Here we reported that hepatic Wilms' tumor 1-associating protein (WTAP) is a key integrative regulator of ectopic lipid accumulation and inflammation during NASH progression. Hepatic deletion of *Wtap* leads to NASH due to the increased lipolysis in white adipose tissue, enhanced hepatic free fatty acids uptake and induced inflammation, all of which are mediated by IGFBP1, CD36 and cytochemokines such as CCL2, respectively. WTAP binds to specific DNA motifs which are enriched in the promoters and suppresses gene expression (e.g., *Igfbp1*, *Cd36* and *Ccl2*) with the involvement of HDAC1. In NASH, WTAP is tranlocated from nucleus to cytosol, which is related to CDK9-mediated phosphorylation. These data uncover a mechanism by which hepatic WTAP regulates ectopic lipid accumulation and inflammation during NASH progression.

## Abstract (web summary)

Ectopic lipid accumulation and inflammation are the essential signs of NASH. Here, the authors show that hepatic WTAP is a key integrative repressor of ectopic lipid accumulation and inflammation during NASH progression, and hepatic deletion of *Wtap* promotes both of them, leading to NASH

## Introduction

The liver plays a key role in metabolic homeostasis including the homeostasis of glucose, lipid, and amino acid metabolism. An imbalance of hepatic metabolic homeostasis will lead to liver diseases. A common chronic liver disease is non-alcoholic fatty liver disease (NAFLD), which ranges from non-alcoholic fatty liver (NAFL) to non-alcoholic steatohepatitis (NASH). NASH is characterized by hepatic steatosis, liver injury, chronic inflammation and liver fibrosis, which is one of the important steps during the pathogenesis of cirrhosis and hepatocellular carcinoma (HCC). During NASH pathogenesis, ectopic lipid accumulation is one of the major contributor to NASH progression. In human NAFLD patients, about 60% of hepatic triglycerides are derived from serum free fatty acids (FFAs), which are mainly from white adipose tissue (released after TAG lipolysis)^1^. The rates of lipolysis in white adipose tissue are increased in NASH patients^2^, and hepatic free fatty acid uptake is also increased in NAFL and NASH due to the increased expression of CD36^3^. In addition, most of patients with lipodystrophy develop severe NAFL and NASH^4,5^. In the last several years, many factors, including SEIPIN/BSCL2, Insulin receptor, AGPAT2, PPARγ, CIDEC, perilipin-1, and AKT-2, in white adipose tissue have been identified to regulate lipodystrophy or lipoatrophy in rodents^4,6–10^. However, whether and how liver affect lipoatrophy, which further contributes to NASH pathogenesis, is not fully understood.

Ectopic lipid accumulation in the liver causes NAFL, while the progression of NAFL to NASH is promoted by persistent steatosis (lipotoxity) and inflammation. It has been reported that chemokines such as CCL2 and its receptor CCR2 are abnormally upregulated in NASH^11,12^, which is known as one of the second hits for NASH progression. However, how ectopic lipid accumulation and inflammation in the liver affect NASH progression in the setting of lipoatrophy is not investigated yet.

Wilms' tumor 1-associating protein (WTAP) is a nuclear protein, which is located in the speckles and partially co-localized with splicing factors^13,14^. Recent studies showed that WTAP can serve as a regulatory subunit of m^6^A writer machine and regulates m^6^A modification and alternative splicing^15,16^. WTAP also regulates many physiological and pathological processes such as X chromosome imprinting^17^, cell proliferation^18^, white adipogenesis^19^ and tumorgenesis^20,21^ by modulating m^6^A modification and RNA alternative splicing. *Wtap* knockout mice were embryonic lethal^22^, indicating that WTAP is essential for development of multiple organs. However, the function of WTAP in metabolic homeostasis is not explored. Whether hepatic WTAP regulates ectopic lipid accumulation and NASH progression is largely unknown.

Here, we have demonstrated that hepatic WTAP integratively regulates lipoatrophy, ectopic lipid accumulation and inflammation during NASH progression. Hepatic deletion of *Wtap* induces the expression and secretion of IGFBP1, which enhances lipolysis in the eWAT and increases serum FFAs, leading to hepatic steatosis. In addition, hepatic *Wtap* deficiency increases the expression of CD36 and CCL2, which enhances hepatic FFAs uptake and inflammation, causing NASH in *Wtap*-HKO mice. Mechanistically, WTAP binds to specific DNA motifs which are enriched in the promoters and suppresses gene expression (e.g., *Igfbp1*, *Cd36* and *Ccl2*) with the involvement of HDAC1. Furthermore, WTAP is translocated from nucleus to cytosol in NASH, which is related to CDK9-mediated phosphorylation. These data uncover a mechanism by which downregulation of nuclear WTAP in hepatocytes induces lipoatrophy and causes NASH *via* a histone modification pathway.

## Results

### WTAP is highly expressed in the liver and hepatic deletion of *Wtap* causes non-alcoholic steatohepatitis (NASH)

**Fig. 1:**
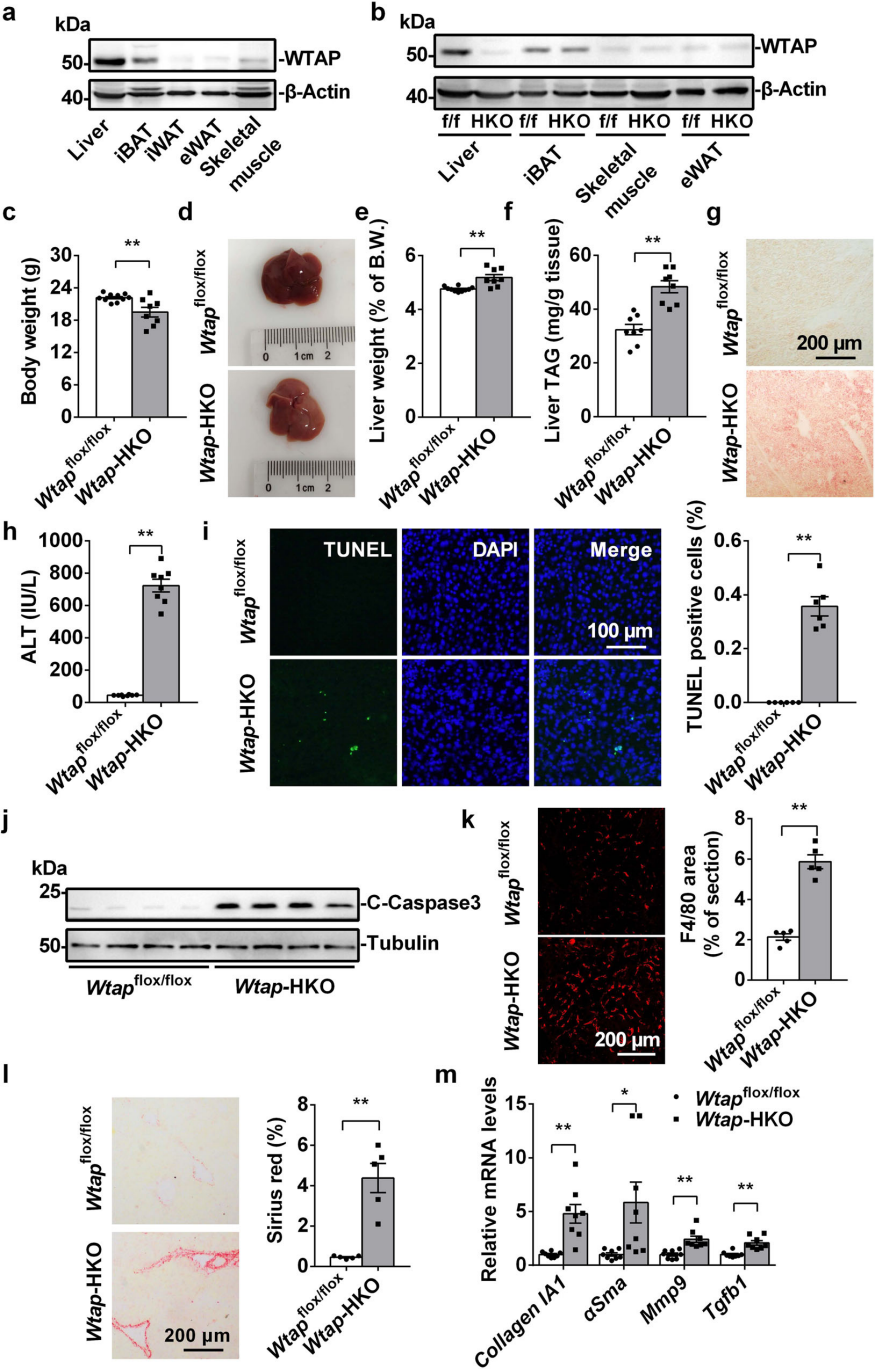
Hepatic deletion of *Wtap* causes non-alcoholic steatohepatitis (NASH) under the normal chow feeding condition. **a** WTAP protein levels in liver, iBAT, iWAT, eWAT and skeletal muscle of WT mice. The samples were derived from the same experiment and the blots were processed in parallel. This experiment was repeated for three times independently with similar results. **b** WTAP protein levels in liver, iBAT, skeletal muscle and eWAT of *Wtap*^flox/flox^ and *Wtap-*HKO mice at 8 weeks old. The samples were derived from the same experiment and the blots were processed in parallel. This experiment was repeated for three times independently with similar results. **c** Body weights of *Wtap*^flox/flox^ and *Wtap*-HKO mice at 8 weeks old under the normal chow feeding condition (*Wtap*^flox/flox^, *n* = 10; *Wtap*-HKO, *n* = 8; *P* = 0.0057). **d** Representative pictures of livers from *Wtap*^flox/flox^ and *Wtap-*HKO mice. **e** Liver weights (*Wtap*^flox/flox^, *n* = 10; *Wtap*-HKO, *n* = 8; *P* = 0.0011). **f** Liver TAG levels (*n* = 8 for each group; *P* < 0.0001). **g** Oil Red O staining was performed in the liver sections from *Wtap*^flox/flox^ and *Wtap-*HKO mice at 8 weeks old (*n* = 5 for each group). n was the number of biologically independent mice. Mice of the same genotype showed similar phenotypes. Representative Oil Red O staining images were shown. **h** Serum ALT activity in *Wtap*^flox/flox^ and *Wtap-*HKO mice at 8 weeks old (*n* = 8 for each group; *P* < 0.0001). **i** The TUNEL-positive cells in liver sections of *Wtap*^flox/flox^ and *Wtap-*HKO mice at 8 weeks old (*n* = 6 for each group; *P* < 0.0001). **j** Cleaved caspase3 levels were measured by immunoblotting (*n* = 4 for each group). The samples were derived from the same experiment and the blots were processed in parallel. This experiments were repeated for three times with similar results. **k** F4/80 immunostaining of liver sections (*n* = 5 for each group; *P* < 0.0001). **l** Sirius Red staining of liver sections (*n* = 5 for each group; *P* = 0.0006). **m** RT-qPCR analysis of mRNA levels (*n* = 8 for each group; *Collagen IA1*, *P* = 0.0063; *αSma*, *P* = 0.0234; *Mmp9*, *P* = 0.0006; *Tgfb1*, *P* = 0.0003). n was the number of biologically independent mice. Data represent the mean ± SEM. Significance was determined by unpaired two-tailed Student’s *t* test analysis. *p < 0.05. **p < 0.01. Source data are provided as a Source Data file.

To determine whether WTAP regulates metabolic homeostasis, we measured the expression of WTAP in metabolic tissues such as liver, interscapular brown adipose tissue (iBAT), skeletal muscle and white adipose tissue. As shown in Fig. 1a, WTAP is highly expressed in the liver and iBAT. To determine whether hepatic WTAP regulates hepatocyte function, hepatocyte-specific *Wtap* knockout mice were generated by crossing *Wtap*^flox/flox^ mice (Supplementary Fig. 1a, b) with *Alb*-Cre mice. The genotype of the *Wtap*-HKO mice is *Wtap*^flox/flox^ *Alb*-Cre^+/−^. As shown in Fig. 1b, WTAP was specifically deleted in the liver of *Wtap*-HKO mice. We did not observe any differences in body weight (Supplementary Fig. 1c), liver weight (Supplementary Fig. 1d), or liver triacylglycerol (TAG) levels (Supplementary Fig. 1e) between *Wtap*^flox/flox^ and *Alb*-Cre mice. Therefore, we used *Wtap*^flox/flox^ mice as the control for *Wtap*-HKO mice in the following experiments. The body weight of *Wtap*-HKO mice was reduced under the normal chow feeding condition (Fig. 1c). The liver showed a light-yellow color (Fig. 1d) and the relative liver weight was increased (Fig. 1e), indicating hepatic deletion of *Wtap* promotes steatosis. To further test this hypothesis, liver TAG levels were measured, and Oil-Red O staining was performed. As shown in Fig. 1f, g, liver TAG levels were significantly increased, and more lipid droplets were seen in *Wtap*-HKO mice under the normal chow feeding condition. These data demonstrate that hepatic deletion of *Wtap* promotes steatosis.

Hepatocyte steatosis may cause liver injury and fibrosis. To test this hypothesis, serum ALT activity, TUNEL-positive cells, cleaved caspase 3, and liver injury-related markers were measured. Serum ALT activities were significantly increased in *Wtap*-HKO mice (Fig. 1h), which suggests that hepatic deletion of *Wtap* causes liver injury and inflammation. We measured hepatocyte apoptosis using TUNEL assays. The number of TUNEL-positive cells was significantly increased in *Wtap*-HKO mice (Fig. 1i). In addition, cleaved caspase 3 levels were much higher in the livers of *Wtap*-HKO mice (Fig. 1j). Hepatocyte death also leaded to immune cell infiltration. F4/80-positive cells were significantly increased in the livers of *Wtap*-HKO mice (Fig. 1k). Moreover, liver sections from *Wtap*-HKO mice contained significantly larger Sirius Red-positive areas than those in *Wtap*^flox/flox^ mice (Fig. 1l). Consistently, the expression of fibrosis markers (*collagen IA1*, *aSma* and *Mmp9*) and profibrogenic factor (*Tgfb1*) was significantly increased in the livers of *Wtap*-HKO mice (Fig. 1m). These data demonstrate that hepatic deletion of *Wtap* results in liver injury and fibrosis.

Next, we tested whether hepatic deletion of *Wtap* also promotes diet-induced NASH. *Wtap*-HKO and *Wtap*^flox/flox^ mice were fed a NASH diet. Short time (5 weeks) feeding with NASH diet was not able to induced NASH in *Wtap*^flox/flox^ control mice. However, *Wtap*-HKO mice displayed severe NASH phenotypes, as revealed by higher liver weight (Supplementary Fig. 2a, b), higher liver TAG levels (Supplementary Fig. 2c), more hepatic lipid droplets (Supplementary Fig. 2d), and higher serum ALT activities (Supplementary Fig. 2e) compared with *Wtap*^flox/flox^ mice. The number of TUNEL-positive cells was significantly increased in *Wtap*-HKO mice (Supplementary Fig. 2f). In addition, cleaved caspase3 was much higher in *Wtap*-HKO mice (Supplementary Fig. 2g). Hepatocyte death also leaded to immune cell infiltration and liver fibrosis. F4/80-positive cells were significantly increased (Supplementary Fig. 2h). Moreover, liver sections from *Wtap*-HKO mice contained significantly larger Sirius Red-positive areas than those in *Wtap*^flox/flox^ mice (Supplementary Fig. 2i). Consistently, the expression of fibrosis markers (*collagen IA1*, *aSma* and *Mmp9*) and profibrogenic factor (*Tgfb1*) was dramatically increased in *Wtap*-HKO mice (Supplementary Fig. 2j). These data indicate that *Wtap*-HKO mice are more sensitive to NASH diet-induced NASH.

### Hepatic deletion of *Wtap* promotes FFAs uptake and inflammation in the liver

**Fig. 2:**
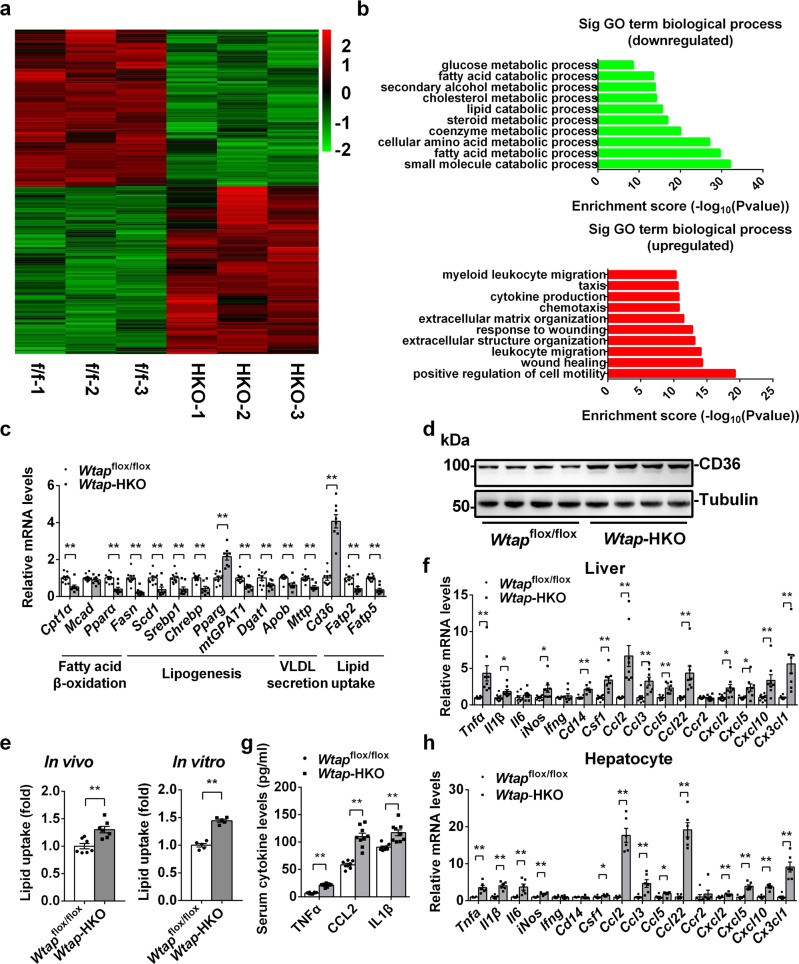
Hepatic deletion of *Wtap* promotes FFAs uptake and inflammation in the liver. **a** RNA-seq analysis was performed in the livers of *Wtap*^flox/flox^ and *Wtap-*HKO mice at 8 weeks old. The differentially expressed genes (DEGs) (HKO VS ff) including 3486 downregulated genes and 3706 upregulated genes were illustrated in a volcanoplot (|log2foldchange| > 0 and pval < 0.05). **b** Top GO biological process terms enriched in downregulated and upregulated genes. **c** RT-qPCR analysis of lipid metabolism related genes in the livers of *Wtap*^flox/flox^ and *Wtap-*HKO mice at 8 weeks old (*Wtap*^flox/flox^, *n* = 10; *Wtap*-HKO, *n* = 8; *Cpt1α*, *P* = 0.0017; *Mcad*, *P* = 0.2985; *Pparα*, *P* = 0.0002; *Fasn*, *P* < 0.0001; *Scd1*, *P* = 0.0016; *Srebp1*, *P* = 0.0012; *Chrebp*, *P* = 0.0002; *Pparg*, *P* < 0.0001; *mtGPAT1*, *P* = 0.0019; *Dgat1*, *P* = 0.0204; *Apob*, *P* = 0.005; *Mttp*, *P* = 0.0003; *Cd36*, *P* < 0.0001; *Fatp2*, *P* = 0.0011; *Fatp5*, *P* < 0.0001). **d** CD36 protein levels in *Wtap*^flox/flox^ and *Wtap-*HKO mice at 8 weeks old were measured by immunoblotting (*n* = 4 for each group). The samples were derived from the same experiment and the blots were processed in parallel. This experiments were repeated for three times with similar results. **e** Relative FFAs uptake levels both in vivo (*n* = 7 for each group; *P* = 0.00197) and in vitro (*n* = 5 for each group; *P* < 0.0001). **f** RT-qPCR analysis of mRNA levels in livers of *Wtap*^flox/flox^ and *Wtap-*HKO mice at 8 weeks old (*n* = 8 for each group; *Tnfα*, *P* = 0.0065; *Il1β*, *P* = 0.01867; *Il6*, *P* = 0.3317; *iNos*, *P* = 0.029; *Infg*, *P* = 0.4315; *Cd14*, *P* < 0.0001; *Csf1*, *P* = 0.00026; *Ccl2*, *P* = 0.00137; *Ccl3*, *P* = 0.0025; *Ccl5*, *P* = 0.0003; *Ccl22*, *P* = 0.0035; *Ccr2*, *P* = 0.7214; *Cxcl2*, *P* = 0.01725; *Cxcl5*, *P* = 0.0147; *Cxcl10*, *P* = 0.0097; *Cx3cl1*, *P* = 0.003). **g** Serum cytokine levels in *Wtap*^flox/flox^ (*n* = 8) and *Wtap-*HKO (*n* = 9) mice at 8 weeks old (TNFα, *P* < 0.0001; CCL2, *P* < 0.0001; IL1β, *P* = 0.0006). **h** RT-qPCR analysis of mRNA levels in primary hepatocytes isolated from *Wtap*^flox/flox^ and *Wtap-*HKO mice at 8 weeks old (*n* = 6 for each group; *Tnfα*, *P* = 0.0014; *Il1β*, *P* < 0.0001; *Il6*, *P* = 0.0078; *iNos*, *P* = 0.0003; *Infg*, *P* = 0.7374; *Cd14*, *P* = 0.7149; *Csf1*, *P* = 0.0336; *Ccl2*, *P* < 0.0001; *Ccl3*, *P* = 0.0067; *Ccl5*, *P* = 0.0281; *Ccl22*, *P* < 0.0001; *Ccr2*, *P* = 0.5374; *Cxcl2*, *P* = 0.0078; *Cxcl5*, *P* < 0.0001; *Cxcl10*, *P* < 0.0001; *Cx3cl1*, *P* = 0.0001). n was the number of biologically independent mice. The cell culture experiments were repeated for three times independently with similar results. Data represent the mean ± SEM. Significance was determined by unpaired two-tailed Student’s *t* test analysis. *p < 0.05. **p < 0.01. Source data are provided as a Source Data file.

To determine the possible mechanisms in *Wtap* deficiency-induced NASH, we performed RNA-seq analysis to detect the changes of gene expression profile in the liver of *Wtap*-HKO mice. As shown in Fig. 2a, 3486 genes were downregulated, whereas 3706 genes were upregulated. Gene ontology (GO) analysis showed that downregulated genes were associated with small molecule catabolic process, fatty acid metabolic process, amino acid metabolic process, steroid metabolic process, cholesterol metabolic process, xenobiotic metabolic process, fatty acid beta-oxidation, alcohol metabolic process, and glucose metabolic process, whereas the upregulated genes were associated with wound healing, leukocyte migration, chemotaxis, and cytokine production (Fig. 2b).

Liver steatosis attributes to an imbalance among free fatty acid uptake, lipogenesis, fatty acid β-oxidation and VLDL secretion^23,24^. RNA-seq and qPCR analysis showed that the expression of genes related to fatty acid β-oxidation (*Cpt1α*, *Mcad* and *Ppara*), lipogenesis (*Fasn*, *Scd1*, *Srebp1*, *Chrebp*, *Pparg*, *mtGPAT1* and *Dgat1*) and VLDL secretion (*ApoB* and *Mttp*) was significantly decreased (Fig. 2a–c). However, *Cd36* (a free fatty acid uptake-related gene) mRNA and protein levels were dramatically increased whereas those of *Fatp2* and *Fatp5* were decreased (Fig. 2c, d), indicating that CD36-mediated FFAs uptake was increased. To further test whether FFAs uptake is increased in *Wtap*-HKO mice, we measured FFA uptake both in vivo and in vitro. As shown in Fig. 2e, acute injection of the fluorescent palmitate analogue BODIPY FL C_16_ into *Wtap*-HKO and *Wtap*^flox/flox^ mice resulted in a significant increase in FFAs uptake by the liver in *Wtap*-HKO mice. To further verify whether hepatic WTAP regulates fatty acid uptake in a cell-autonomous manner, primary hepatocytes were isolated from *Wtap*-HKO and *Wtap*^flox/flox^ mice, and BODIPY FL C_16_ uptake experiments were performed. As shown in Fig. 2e, fatty acid uptake was significantly increased according to the measurement of intracellular fluorescence of BODIPY FL C_16_. These data demonstrate that increased CD36-mediated FFA uptake contributes to the increased liver steatosis seen in *Wtap*-HKO mice.

GO analysis showed that the upregulated genes were associated with wound healing, leukocyte migration, chemotaxis, and positive regulation of cytokine production. qPCR analysis further confirmed these data. As shown in Fig. 2f and Supplementary Fig. 2k, *Tnfa*, *Il1β*, *iNos*, *Cd14*, *Csf1*, *Ccl2*, *Ccl3*, *Ccl5*, *Ccl22*, *Cxcl2*, *Cxcl5*, *Cxcl10* and *Cx3cr1* mRNA levels were significantly increased in *Wtap*-HKO mice under both normal chow and NASH diet feeding conditions. TNFa, IL1β and CCL2 have been shown to promote liver inflammation^11,25,26^. We then measured serum TNFa, IL1β and CCL2 levels. As shown in Fig. 2g and Supplementary Fig. 2l, serum TNFa, IL1β and CCL2 levels were significantly increased in *Wtap*-HKO mice under both normal chow and NASH diet feeding conditions. To further determine whether hepatic WTAP regulates the expression of inflammatory genes in a cell-autonomous manner, primary hepatocytes were isolated from *Wtap*-HKO and *Wtap*^flox/flox^ mice and gene expression was measured by RT-qPCR. As shown in Fig. 2h, *Tnfa*, *Il1β*, *iNos*, *Csf1*, *Ccl2*, *Ccl3*, *Ccl5*, *Ccl22*, *Cxcl2*, *Cxcl5*, *Cxcl10* and *Cx3cr1* mRNA levels were significantly increased in the isolated hepatocytes from *Wtap*-HKO mice. *Ccl2* and *Ccl22* were the top upregulated genes in primary hepatocytes isolated from *Wtap-*HKO mice. These data suggest that hepatic upregulation of cytochemokines such as CCL2 in *Wtap-*HKO mice contributes to more severe NASH.

GO analysis indicated that glucose metabolic process-related genes were downregulated (Fig. 2b). We thus then measured hepatic glucose homeostasis in *Wtap*-HKO and *Wtap*^flox/flox^ mice. It was shown that fasting blood glucose was 21.8% lower in *Wtap*-HKO mice compared to the *Wtap*^flox/flox^ mice, and randomly feeding blood glucose levels were also reduced by 33.1% in these mice under randomly feeding conditions (Supplementary Fig. 3a, b). Hepatic gluconeogenesis was impaired, as revealed by decreased glucose levels after injection of glucose and pyruvate (Supplementary Fig. 3c, d), respectively, and by decreased expression of gluconeogenesis-related genes (*G6pase* and *Pepck*) (Supplementary Fig. 3e). We also noted that other glucose metabolism-related genes such as *Glut2*, *Gys1*, *Pgc1α* and *Hnf4α* were downregulated in *Wtap*-HKO mice (Supplementary Fig. 3e), which caused a reduction of liver glycogen levels in *Wtap*-HKO mice (Supplementary Fig. 3f). These data suggest that hepatic deletion of *Wtap* impairs glucose homeostasis.

### Hepatic deletion of *Wtap* promotes lipolysis in the eWAT

**Fig. 3:**
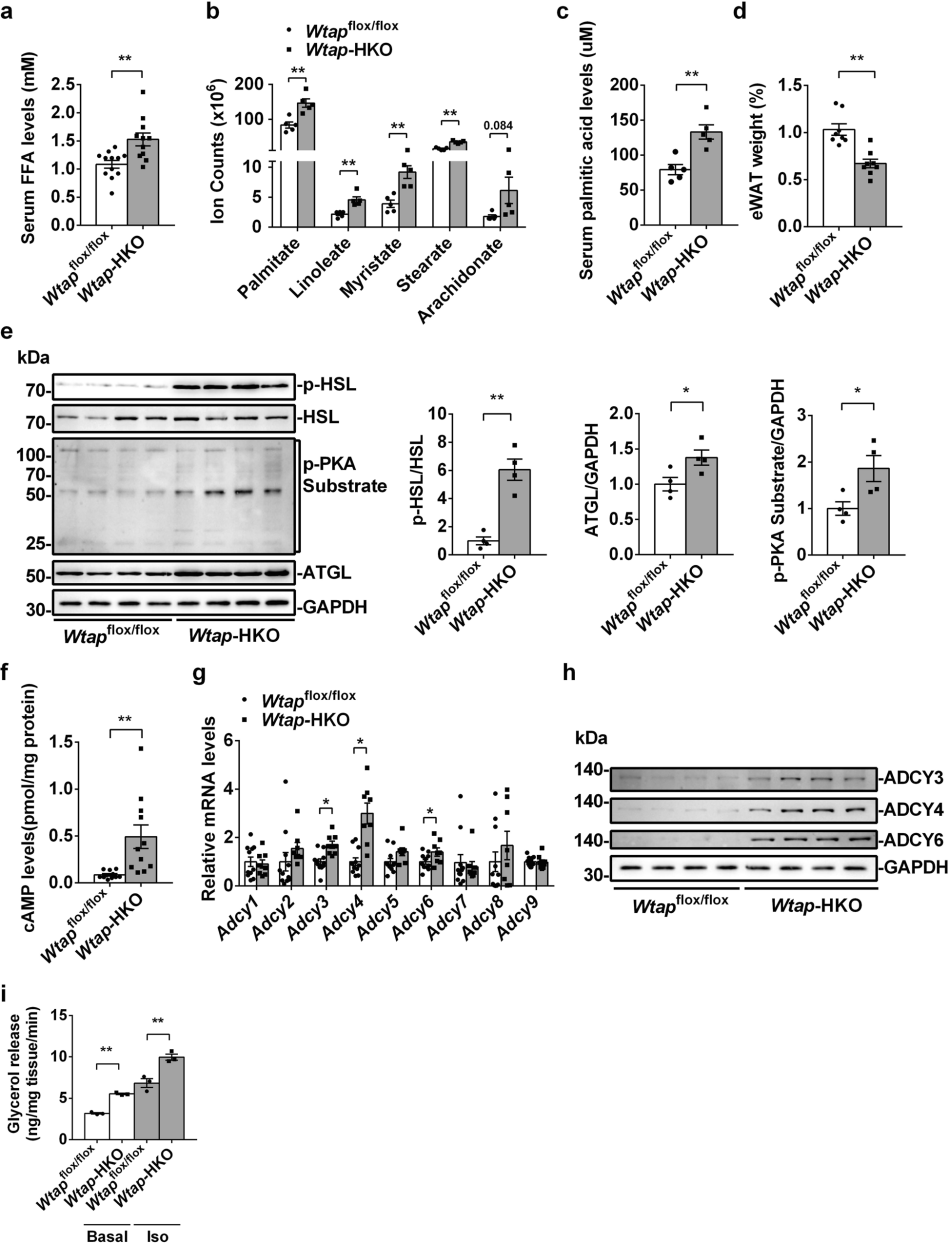
Liver-specific knockout of *Wtap* promotes lipolysis in eWAT. **a** Serum FFA levels in *Wtap*^flox/flox^ and *Wtap-*HKO mice at 8 weeks old (*Wtap*^flox/flox^, *n* = 12; *Wtap*-HKO, *n* = 11; *P* = 0.0028). **b** Relative serum palmitate, linoleate, myristate, stearate and arachidonate levels (*n* = 5 for each group; palmitate, *P* = 0.0028; linoleate, *P* = 0.0019; myristate, *P* = 0.0026; stearate, *P* < 0.0001; arachidonate, *P* = 0.08377). **c** Serum palmitic acid levels (*n* = 5 for each group; *P* = 0.0026). **d** The relative eWAT weights in *Wtap*^flox/flox^ and *Wtap-*HKO mice at 8 weeks old (*n* = 8 for each group; *P* = 0.0003). **e** p-HSL, HSL, p-PKA substrate, ATGL and GAPDH protein levels were measured by immunoblotting in the eWAT of *Wtap*^flox/flox^ and *Wtap-*HKO mice (*n* = 4 for each group). p-HSL, p-PKA substrate and ATGL protein levels were quantified by Image J (*n* = 4 for each group; p-HSL/HSL, *P* = 0.00077; ATGL/GAPDH, *P* = 0.039; p-PKA substrate/GAPDH, *P* = 0.03358). The samples were derived from the same experiment and the blots were processed in parallel. **f** cAMP levels in eWAT of *Wtap*^flox/flox^ and *Wtap-*HKO mice at 8 weeks old (*n* = 11 for each group; *P* = 0.0044). **g** RT-qPCR analysis of Adcys mRNA levels in eWAT of *Wtap*^flox/flox^ and *Wtap-*HKO mice at 8 weeks old (*Wtap*^flox/flox^, *n* = 11; *Wtap*-HKO, *n* = 8; *Adcy1*, *P* = 0.6368; *Adcy2*, *P* = 0.2823; *Adcy3*, *P* = 0.00068; *Adcy4*, *P* = 0.00028; *Adcy5*, *P* = 0.1098; *Adcy6*, *P* = 0.0254; *Adcy7*, *P* = 0.6631; *Adcy8*, *P* = 0.2374; *Adcy9*, *P* = 0.8768). **h** ADCY3, ADCY4 and ADCY6 protein levels in eWAT of *Wtap*^flox/flox^ and *Wtap-*HKO mice at 8 weeks old (*n* = 4 for each group). The samples were derived from the same experiment and the blots were processed in parallel. **i** Ex vivo lipolysis assay (Glycerol release) in the eWAT treated with or without isoproterenol (120 nM) for 4 hours (*n* = 3 for each group; Basal, *P* < 0.0001; Iso, *P* = 0.0085). This experiment was repeated for three times independently. n was the number of biologically independent mice. Data represent the mean ± SEM. Significance was determined by unpaired two-tailed Student’s *t* test analysis. *p < 0.05. **p < 0.01. Source data are provided as a Source Data file.

Increased CD36-mediated FFAs uptake contributes to NASH progression. *Wtap-*HKO mice displayed NASH under the normal chow feeding condition. We then measured serum FFAs levels in *Wtap-*HKO mice. As shown in Fig. 3a, serum FFAs levels were significantly increased in *Wtap-*HKO mice. To determine which FFA was elevated in *Wtap-*HKO mice, serum major FFAs were measured by LC-MS/MS analysis. As shown in Fig. 3b, relative serum palmitate, linoleate, myristate and stearate levels were significantly increased in *Wtap-*HKO mice. Serum palmitic acid levels were elevated by 1.68-fold in *Wtap-*HKO mice (Fig. 3c). These elevated FFAs promoted NASH progression in *Wtap-*HKO mice. Next, we tested whether elevated serum FFAs came from lipolysis of white adipose tissue. We noticed that the epididymal white adipose tissue (eWAT) but not iWAT became smaller in *Wtap-*HKO mice (Fig. 3d and Supplementary Fig. 4a), which may be due to increased lipolysis in eWAT. We then measured the lipolysis-related signaling pathway including p-HSL, HSL, p-PKA substrate and ATGL protein levels in eWAT. As shown in Fig. 3e and Supplementary Fig. 4b, p-HSL, p-PKA substrate and ATGL protein levels were significantly increased in the eWAT but not iWAT of *Wtap-*HKO mice. To further test how PKA signaling is activated in the eWAT of *Wtap-*HKO mice, we measured cAMP levels and the expression of adenylate cyclases (ADCYs). As shown in Fig. 3f, cAMP levels were dramatically increased by 5.66-fold in the eWAT of *Wtap-*HKO mice. *Adcy3*, *Adcy4* and *Adcy6* mRNA levels were significantly elevated, whereas *Adcy1*, *Adcy2*, *Adcy5*, *Adcy7*, *Adcy8* and *Adcy9* mRNA levels were not changed in the eWAT of *Wtap-*HKO mice (Fig. 3g). ADCY3, ADCY4 and ADCY6 protein levels were also significantly increased (Fig. 3h). *Wtap-*HKO mice fed with NASH diet for 5 weeks also showed higher serum FFAs levels, smaller eWAT, and higher lipolysis rate in the eWAT, which are likely due to the increased protein levels of ADCY3, ADCY4, ADCY6, p-HSL, p-PKA substrate and ATGL (Supplementary Fig. 5a–d). In addition, we performed lipolysis assay ex vivo. The lipolysis (glycerol release) in the eWAT of *Wtap*-HKO mice was significantly increased at both basal and isoproterenol treated conditions (Fig. 3i). These data indicate that increased lipolysis in eWAT leads to an induction of serum FFAs in *Wtap*-HKO mice.

### IGFBP1 mediates the induction of lipolysis in eWAT, which contributes to NASH pathogenesis in *Wtap-*HKO mice

**Fig. 4:**
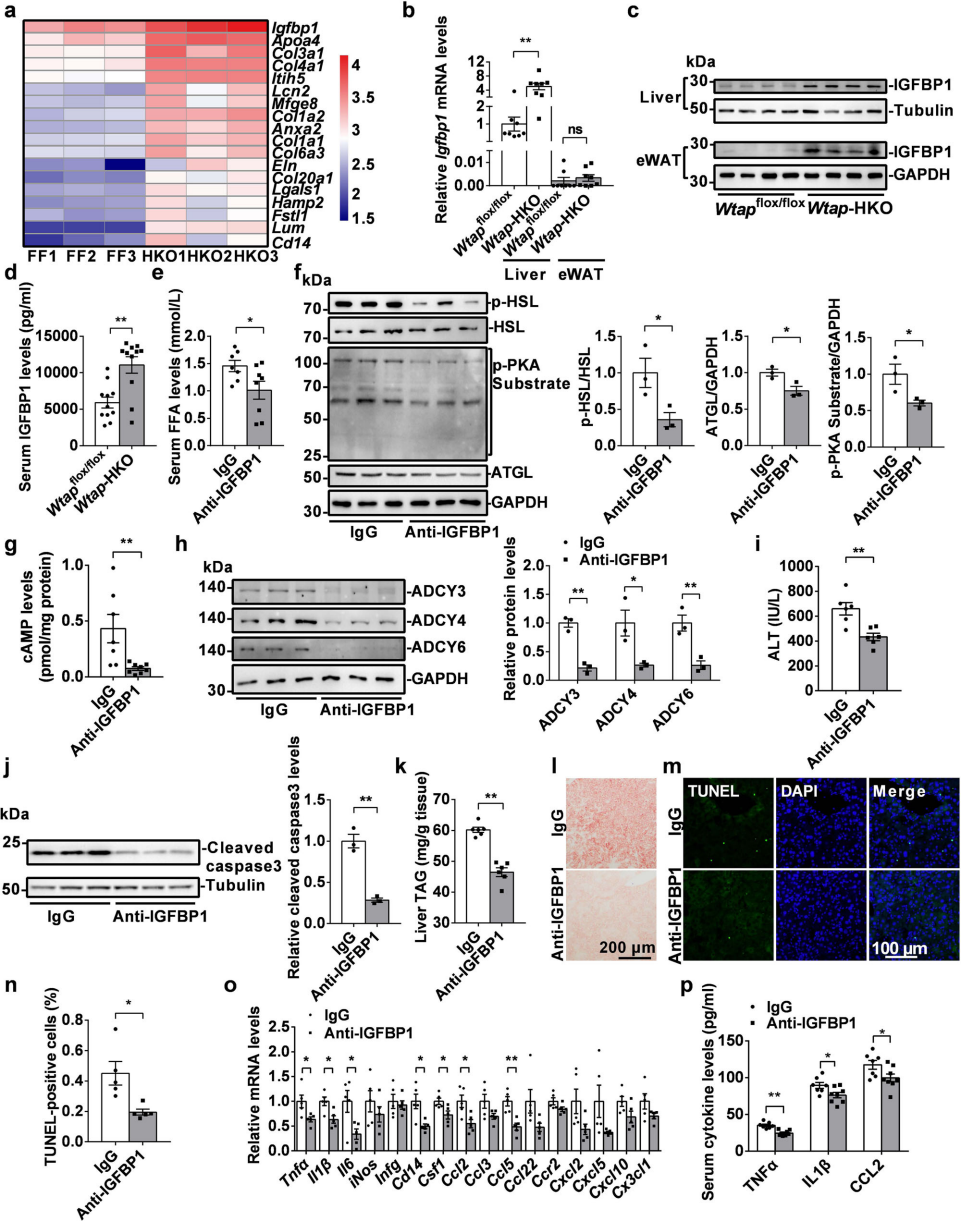
IGFBP1 mediates the induction of lipolysis and NASH in *Wtap-*HKO mice. **a** Heatmap of top differentiated genes encoding secreted proteins in the livers of *Wtap*^flox/flox^ and *Wtap-*HKO mice (*Wtap-*HKO fpkm > 500 and FoldChange > 4.5). Data were presented as log 10 fpkm (*n* = 3 for each group). **b** Relative *Igfbp1* mRNA levels in the liver and eWAT (*n* = 8 for each group; Liver, *P* = 0.0014; eWAT, *P* = 0.547). **c** IGFBP1 protein levels in the liver and eWAT were measured by immunoblotting (*n* = 4 for each group). The samples were derived from the same experiment and the blots were processed in parallel. **d** Serum IGFBP1 protein levels were measured by ELISA (*n* = 11 for each group; *P* = 0.0011). **e**–**p** *Wtap-*HKO mice at 7 weeks old were intravenously injected with a control antibody (IgG) and an anti-IGFBP1 neutralizing antibody per day, respectively, for 5 days. Serum FFA levels were measured (**e**) (IgG, *n* = 7; Anti-IGFBP1, *n* = 8; *P* = 0.0455). p-HSL, HSL, p-PKA substrate, ATGL and GAPDH protein levels in eWAT were measured by immunoblotting and quantified by Image J (**f**) (*n* = 3 for each group; p-HSL/HSL, *P* = 0.0445; ATGL/GAPDH, *P* = 0.0335; p-PKA substrate/GAPDH, *P* = 0.0499). cAMP levels were measured by ELISA (**g**) (IgG, *n* = 7; Anti-IGFBP1, *n* = 8; *P* = 0.00998). The ADCY3, ADCY4 and ADCY6 protein levels in the eWAT were measured by immunoblotting and quantified by Image J (**h**) (*n* = 3 for each group; ADCY3, *P* = 0.0009; ADCY4, *P* = 0.0318; ADCY6, *P* = 0.00918). serum ALT activity were measured (**i**) (*n* = 6 for each group; *P* = 0.0031). Cleaved caspase 3 levels (**j**) (*n* = 3 for each group; *P* = 0.0012), liver TAG levels (**k**) (*n* = 6 for each group; *P* < 0.0001), hepatic lipid droplets (**l**), TUNEL-positive cells (**m**, **n**) (*n* = 5 for each group; *P* = 0.0129), and cytochemokine mRNA levels (**o**) (*n* = 5 for each group; *Tnfα*, *P* = 0.0294; *Il1β*, *P* = 0.0133; *Il6*, *P* = 0.0267; *iNos*, *P* = 0.3452; *Infg*, *P* = 0.6684; *Cd14*, *P* = 0.0111; *Csf1*, *P* = 0.0292; *Ccl2*, *P* = 0.0172; *Ccl3*, *P* = 0.0824; *Ccl5*, *P* = 0.0025; *Ccl22*, *P* = 0.0722; *Ccr2*, *P* = 0.0969; *Cxcl2*, *P* = 0.0693; *Cxcl5*, *P* = 0.092; *Cxcl10*, *P* = 0.0747; *Cx3cl1*, *P* = 0.1067) were measured in the liver. Serum cytokine levels were measured by ELISA (**p**) (IgG, *n* = 7; Anti-IGFBP1, *n* = 8; TNFα, *P* = 0.000178; IL1β, *P* = 0.0323; CCL2, *P* = 0.0456). For immunoblotting, the samples were derived from the same experiment and the blots were processed in parallel. n was the number of biologically independent mice. Data represent the mean ± SEM. Significance was determined by unpaired two-tailed Student’s *t* test analysis. *p < 0.05. **p < 0.01. Source data are provided as a Source Data file.

Hepatic deletion of *Wtap* may promote expression and secretion of secreted factors that further induce lipolysis in eWAT. To investigate which secreted proteins in the liver of *Wtap*-HKO mice promote lipolysis in eWAT, we analyzed the mouse secretome gene set between *Wtap*-HKO and *Wtap*^flox/flox^ mice. The secretome database contains 1819 mouse genes predicted to encode secreted proteins^27^. As shown in Fig. 4a, 18 genes were highly expressed (fpkm > 500) and significantly increased (FoldChange > 4.5). Among these genes, *Igfbp1* is the highest expressed gene. IGFBP1 is a liver-specific expressed and secreted protein^28^. qPCR data showed that *Igfbp1* was specifically expressed in the liver and significantly increased in the liver but not the eWAT of *Wtap*-HKO mice (Fig. 4b). Immunoblotting showed that IGFBP1 levels were dramatically increased in both liver and eWAT but not iWAT of *Wtap*-HKO mice (Fig. 4c and Supplementary Fig. 4b). ELISA data showed that serum IGFBP1 levels were also significantly increased in both normal chow- and NASH diet-feeding *Wtap*-HKO mice (Fig. 4d and Supplementary Fig. 5e). *Igfbp1* mRNA levels in eWAT of *Wtap*-HKO and *Wtap*^flox/flox^ mice were much lower (CT value ≥ 33, some of them showed no CT value) than that in the liver (CT value ≤ 22) (Fig. 4b). These data indicate that hepatic deletion of *Wtap* leads to higher expression and secretion of IGFBP1 in the liver, which goes into the eWAT of *Wtap*-HKO mice.

In human patients with NASH and MCD-induced NASH mouse model, the higher lipolysis rate in WAT has been reported^2,29^. It has been reported that serum IGFBP1 levels were significantly increased in human patients with severe NASH (advanced fibrosis in NAFLD)^30^. We also observed that serum IGFBP1 levels were dramatically elevated in MCD-induced NASH (Supplementary Fig. 6a). The IGFBP1 levels and the lipolysis signaling pathway (p-PKA substrate/p-HSL/ATGL) were significantly increased in the eWAT of MCD-feeding mice (Supplementary Fig. 6b). These data suggest that IGFBP1 is associated with the enhanced lipolysis in eWAT during NASH progression.

Next, we asked whether IGFBP1 is essential for the elevated lipolysis in the eWAT of *Wtap*-HKO mice. IGFBP1 was neutralized by an anti-IGFBP1 antibody in *Wtap*-HKO mice. As shown in Fig. 4e, f, neutralization of IGFBP1 prevented against the elevated lipolysis in the white adipose tissue, as revealed by decreased serum FFAs (Fig. 4e), and decreased p-HSL, p-PKA substrate and ATGL protein levels (Fig. 4f). This phenotype was likely due to the dramatically decreased cAMP levels (Fig. 4g) and the reduced expression of ADCY3, ADCY4 and ADCY6 (Fig. 4h). Ex vivo incubation with the anti-IGFBP1 antibody in eWAT of *Wtap*-HKO mice also showed a reduction of lipolysis, as revealed by decreased glycerol release (Supplementary Fig. 7a), and decreased p-HSL, p-PKA substrate and ATGL protein levels (Supplementary Fig. 7b). IGFBP1 was able to increase lipolysis in eWAT of *Wtap*^flox/flox^, *Wtap*^flox/−^ *Alb*-Cre+(half deletion of *Wtap* in the liver), and *Wtap*^flox/flox^ *Alb*-Cre (*Wtap*-HKO) mice in a dose-dependent manner (Supplementary Fig. 8a). Although *Wtap*^flox/−^ *Alb*-Cre+ mice displayed half deletion of *Wtap* in the liver (Supplementary Fig. 8c), IGFBP1 induced lipolysis in eWAT of *Wtap*^flox/−^ *Alb*-Cre+ mice was similar with that in *Wtap*^flox/flox^ mice (Supplementary Fig. 8a). This was likely because half deletion of *Watp* did not increase the expression of IGFBP1 (Supplementary Fig. 8c). As expected, the basal lipolysis was much higher in eWAT of *Wtap*-HKO mice (Supplementary Fig. 8a, b). However, the induction rate (k value) of lipolysis induced by different dose of IGFBP1 was much lower in eWAT of *Wtap*-HKO mice (Supplementary Fig. 8a). This was likely because higher levels of hepatic IGFBP1 already bound to the adipocytes in eWAT of *Wtap*-HKO mice (Supplementary Fig. 8c). Furthermore, IGFBP1 promoted lipolysis in primary adipocytes (Supplementary Fig. 9a), which was likely due to the increased PKA signaling pathway (Supplementary Fig. 9b). IGFBP1 is a part of the growth hormone (GH) and insulin-like growth factor 1 (IGF1) axis^31^. GH is mainly expressed in pituitary gland. Hepatocyte-specific deletion of *Wtap* did not change the expression of *GH* and *Wtap* in pituitary gland (Supplementary Fig. 10a). RNA-seq data (GSE168850) showed that *GH* was not expressed in the livers of *Wtap*^flox/flox^ and *Wtap*-HKO mice. These data indicate that GH unlikely regulates IGFBP1 expression in *Wtap*-HKO mice. RNA-seq analysis (GSE168850) and RT-qPCR showed that *Igf1* mRNA levels were significantly decreased in *Wtap*-HKO mice (Supplementary Fig. 10b). Serum IGF1 levels were slightly decreased by 28.8% (Supplementary Fig. 10c). However, it has been reported that liver-specific deletion of *Igf1*, which causes a 75% reduction in circulating IGF1, does not affect lipolysis in white adipose tissue^32^. Therefore, GH/IGF1 axis unlikely contributes to the increased lipolysis in the eWAT of *Wtap*-HKO mice. These data demonstrate that hepatic deletion of *Wtap* promotes lipolysis in the white adipose tissue by secreting more IGFBP1 independent of GH/IGF1 axis.

Interestingly, neutralization of IGFBP1 prevented against NASH in *Wtap-*HKO mice, as revealed by lower serum ALT activity (Fig. 4i), less cleavage of caspase 3 (Fig. 4j), lower liver TAG levels (Fig. 4k), fewer hepatic lipid droplets (Fig. 4l) and less TUNEL-positive cells (Fig. 4m, n). The expression of cytokines such as *Tnfa*, *Il1β*, *Il6*, *Cd14*, *Csf1*, *Ccl2*, and *Ccl5* was significantly downregulated in IGFBP1 neutralizing *Wtap-*HKO mice (Fig. 4o). Serum TNFa, IL1β and CCL2 levels were also significantly reduced in IGFBP1 neutralizing *Wtap*-HKO mice (Fig. 4p). These data demonstrate that neutralization of IGFBP1 prevents against NASH progression in *Wtap-*HKO mice, and further support that increased IGFBP1 may be the primary cause for the increased lipolysis in the white adipose tissue and NASH in *Wtap-*HKO mice.

### WTAP regulates transcription of *Igfbp1*, *Cd36* and *Ccl2*

Since increased expression of IGFBP1, CD36 and cytochemokines such as CCL2 promotes NASH progression in *Wtap-*HKO mice, we next asked how WTAP regulated their expression. WTAP has been shown to regulate RNA alternative splicing (AS), we checked the RNA-seq data and found that 344 transcripts displayed significant AS changes (Supplementary Data 1). Unfortunately, we did not find *Igfbp1*, *Cd36* and *Ccl2* transcripts in this list (Supplementary Data 1). WTAP interacts with METTL3 as a regulatory subunit of m^6^A writer machine and regulates m^6^A modification^15,16^. To determine whether WTAP regulates m^6^A modification and further regulates the expression of *Igfbp1*, *Cd36* and *Ccl2*, m^6^A RNA immunoprecipitation sequencing (m^6^ARIP-seq) analysis was performed in the livers of *Wtap*-HKO and *Wtap*^flox/flox^ mice. Consistent with published m^6^ARIP-seq results^33,34^, the m^6^A peaks identified in the livers of *Wtap*^flox/flox^ mice were enriched at the stop codon and 3´-UTR, and the canonical GGACU motif was identified (Supplementary Fig. 11a, b). However, the m^6^A peaks in the livers of *Wtap*-HKO mice were dispersed at the 5´-UTR and stop codon, and the canonical GGACU motif was not found (Supplementary Fig. 11a). In the livers of *Wtap*^flox/flox^ mice, m^6^ARIP-seq analysis showed that transcripts with significant m^6^A peaks came from 9020 genes (Supplementary Data 2). Transcripts with significantly decreased m^6^A levels in the livers of *Wtap*-HKO mice came from 2314 genes (Supplementary Fig. 11c). GO analysis showed that the transcripts with downregulated m^6^A peaks were related to metabolic process, cellular metabolic process, organonitrogen metabolic process and cellular catabolic process (Supplementary Fig. 11d). KEGG pathway analysis showed that the transcripts with downregulated m^6^A peaks were associated with metabolic pathways, protein processing in endoplasmic reticulum, peroxisome, AMPK signaling pathway, fatty acid metabolism, porphyrin and chlorophyll metabolism, biosynthesis of unsaturated fatty acids, and RNA transport (Supplementary Fig. 12). However, no significant m^6^A peaks in the *Igfbp1*, *Cd36* or *Ccl2* transcript were detected in the livers of *Wtap*^flox/flox^ mice (Supplementary Data 2). *Igfbp1*, *Cd36* and *Ccl2* were not present in the gene/transcript list with differential m^6^A peaks (Supplementary Fig. 11c). These data indicate that WTAP regulates the expression of *Igfbp1*, *Cd36* or *Ccl2*, but that it is unlikely due to an m^6^A modification in their transcripts.

**Fig. 5:**
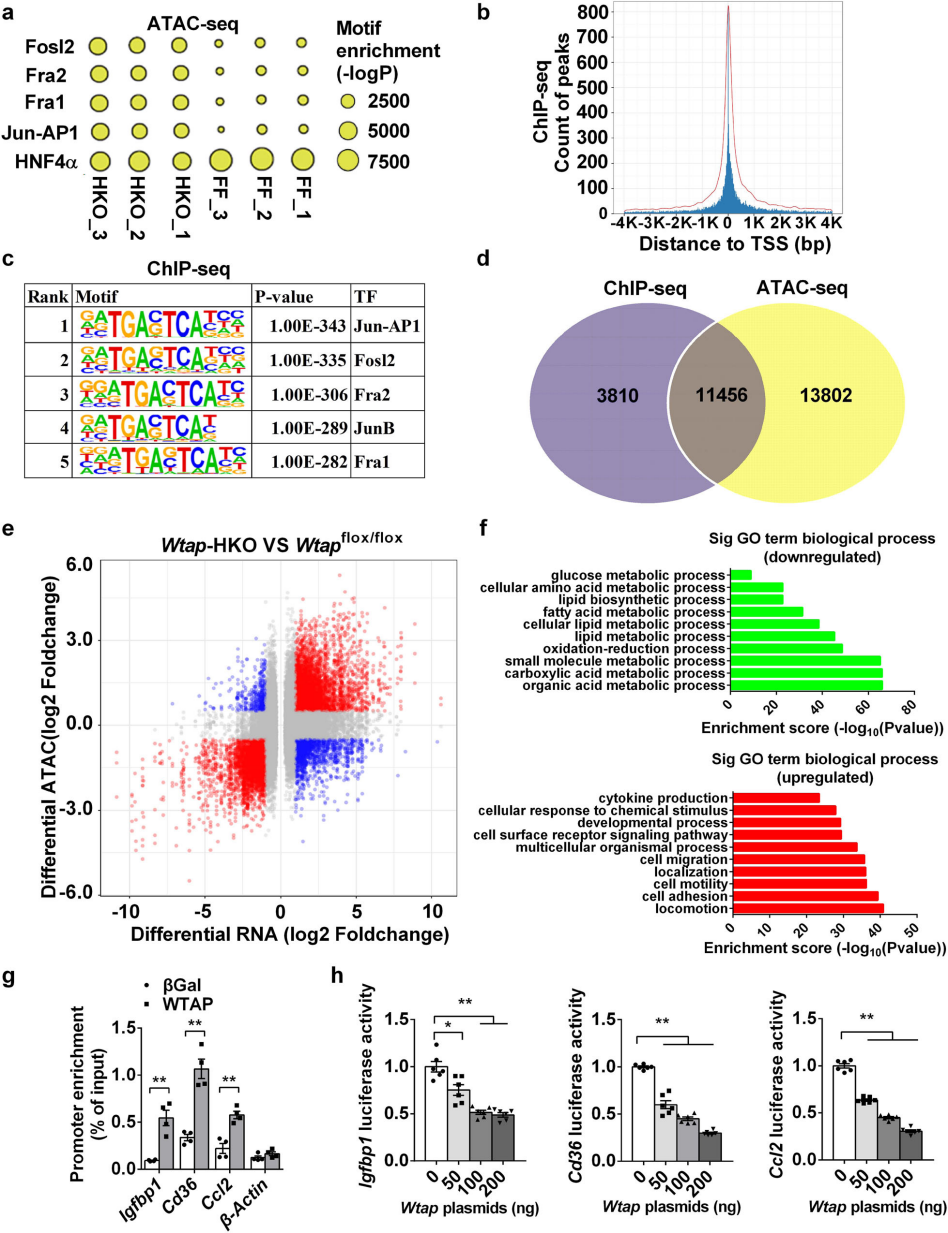
WTAP binds to the gene promoters and regulates *Igfbp1*, *Cd36* and *Ccl2* transcription. **a** Significantly differential transcription factor (TF) binding motifs were identified by analyzing ATAC-seq data collected from livers of *Wtap-*HKO and *Wtap*^flox/flox^ mice at 8-week old (*n* = 3 for each group). **b** Primary hepatocytes were infected with Ad-FLAG-WTAP adenovirus. The hepatocyte nuclei samples were immunoprecipitated with FLAG beads. Immunoprecipitated DNA was extracted for sequencing. Distance of peaks to TSS was shown. **c** Motif analysis of ChIP-seq data showed that the consensus of DNA binding motif was TGASTCA. The top five DNA binding motifs were related to transcription factors Jun-AP1, Fosl2, Fra2, JunB, and Fra1. **d** Combined analysis of ChIP-seq and ATAC-seq identified 11,456 common genes, which were illustrated in a venn diagram plot. **e** ATAC-seq and RNA-seq was performed in the livers of *Wtap*^flox/flox^ and *Wtap-*HKO mice at 8 weeks old. Combined analysis of ATAC-seq and RNA-seq identified the differentially expressed genes (DEGs) (HKO VS ff) including 953 downregulated genes and 1532 upregulated genes, which were illustrated in a volcanoplot (*n* = 3 for each group). **f** Top GO biological process terms enriched in downregulated and upregulated genes. **g** Binding of FLAG-WTAP to the promoters of *Igfbp1*, *Cd36*, *Ccl2* and *β-Actin* were assessed in the primary hepatocytes infected with Ad-FLAG-WTAP or βGal adenovirus by ChIP-qPCR. The hepatocyte samples were immunoprecipitated with FLAG beads. Immunoprecipitated DNA was extracted for qPCR analysis (*n* = 4 for each group; *Igfbp1*, *P* = 0.0014; *Cd36*, *P* = 0.0005; *Ccl2*, *P* = 0.0016; *β-Actin*, *P* = 0.1846). **h** *Igfbp1*, *Cd36* or *Ccl2* promoter luciferase reporter plasmids were cotransfected with WTAP or empty expression vector by polyethylenimine (Sigma) into HEK293T cells. Twenty-four hours later, HEK293T cells were lysed in reporter lysis buffer, and luciferase activity was measured and normalized to β-Gal activity (*n* = 6 for each group; *Igfbp1* 0 VS 50 *P* < 0.0107, others *P* < 0.0001; *Cd36*, *P* < 0.0001; *Ccl2*, *P* < 0.0001). n was the number of biologically independent mice or cell samples. The cell culture experiments were repeated for three times independently with similar results. Data represent the mean ± SEM. Significance was determined by unpaired two-tailed Student’s *t* test analysis. *p < 0.05. **p < 0.01. Source data are provided as a Source Data file.

It has been reported that RNA binding proteins also regulate chromatin accessibility and gene transcription^35,36^. To further test this possibility, we mapped open chromatin in the liver of *Wtap-*HKO mice using assay for transposase-accessible chromatin-sequencing (ATAC-seq). As expected, we observed strong enrichment of open chromatin in the gene promoters and the ATAC-seq peaks near the TSS (Supplementary Fig. 13a, b). ATAC-seq data analysis identified 15,903 upregulated peak-related genes and 16,042 downregulated peak-related genes (Supplementary Data 3). Next, using motif analysis software HOMER, we found several transcription factor (TF) binding motifs. The binding motifs of four TFs (Fosl2, Fra1/2, Jun-AP1) were differentially open in the livers of *Wtap*-HKO mice, whereas the binding motifs of one TF (NHF4α) differentially close in the livers of *Wtap*-HKO mice (Fig. 5a). To further test whether WTAP directly binds to the promoters, ChIP-sequencing was performed in FLAG-WATP-overexpressing hepatocytes. ChIP-seq analysis identified 15,266 genes with peaks (Supplementary Data 4). Most of peaks were located near the TSS (Fig. 5b). 58.91% of peaks were located in the promoters (Supplementary Fig. 14). Motif analysis showed that the consensus of DNA binding motif was TGASTCA (Fig. 5c). The top five DNA binding motifs were related to transcription factors Jun-AP1, Fosl2, Fra2, JunB, and Fra1 (Fig. 5c). Interestingly, these transcription factors were also associated with the significantly upregulated peak-related genes in *Wtap*-HKO mice by mapping the open chromatin using ATAC-seq (Fig. 5a). Combined analysis of ChIP-seq and ATAC-seq identified 11,456 common genes (Fig. 5d). These data indicate that WTAP directly binds to the promoters and regulates chromatin accessibility in the liver.

Combined analysis of ATAC-seq and RNA-seq data showed that 953 genes were downregulated in these two datasets, whereas 1532 genes were upregulated (Fig. 5e and Supplementary Data 5). GO analysis showed that downregulated genes were associated with organic acid metabolic process, carboxylic acid metabolic process, small molecular metabolic process, oxidation-reduction process, lipid metabolic process, fatty acid metabolic process, lipid biosynthetic process, cellular amino acid metabolic process, and glucose metabolic process, whereas the upregulated genes were associated with locomotion, cell adhesion, cell motility, localization, cell migration, multicellular organismal process, cell surface receptor signaling pathway, developmental process, cellular response to chemical stimulus, and cytokine production (Fig. 5f). The KEGG analysis showed that the downregulated genes were associated with metabolic pathways, glycolysis/gluconeogenesis, and PPAR signaling pathway (Supplementary Fig. 15), whereas the upregulated genes were associated with cytokine-cytokine receptor interaction, ECM-receptor interaction, pathways in cancer, regulation of actin cytoskeleton, phagosome, and PI3K-Akt signaling pathways (Supplementary Fig. 16). The upregulated genes in both ATAC-seq and RNA-seq analysis included *Igfbp1*, *Cd36* and *Ccl2* (Fig. 5e and Supplementary Data 5), which indicates that deletion of *Wtap* promotes *Igfbp1*, *Cd36* and *Ccl2* transcription. Furthermore, WTAP chromatin immunoprecipitation (ChIP)-seq analysis showed that *Igfbp1*, *Cd36* and *Ccl2* were in the list of genes with peaks (Supplementary Data 4). Consistently, WTAP binds to the *Igfbp1*, *Cd36* and *Ccl2* promoters but does not bind to the *β-Actin* (*Actb)* promoter in the FLAG-WTAP*-*overexpressing hepatocytes, as detected by chromatin immunoprecipitation quantitative PCR (ChIP-qPCR) (Fig. 5g). In addition, promoter luciferase assays showed that WTAP decreased *Igfbp1*, *Cd36* and *Ccl2* promoter luciferase activities in a dose-dependent manner (Fig. 5h). These data demonstrate that WTAP serves as a transcriptional repressor of the *Igfbp1*, *Cd36* and *Ccl2* genes.

### WTAP interacts with HDAC1 and regulates H3K9ac and H3K27ac levels in the promoters of *Igfbp1*, *Cd36* and *Ccl2*

**Fig. 6:**
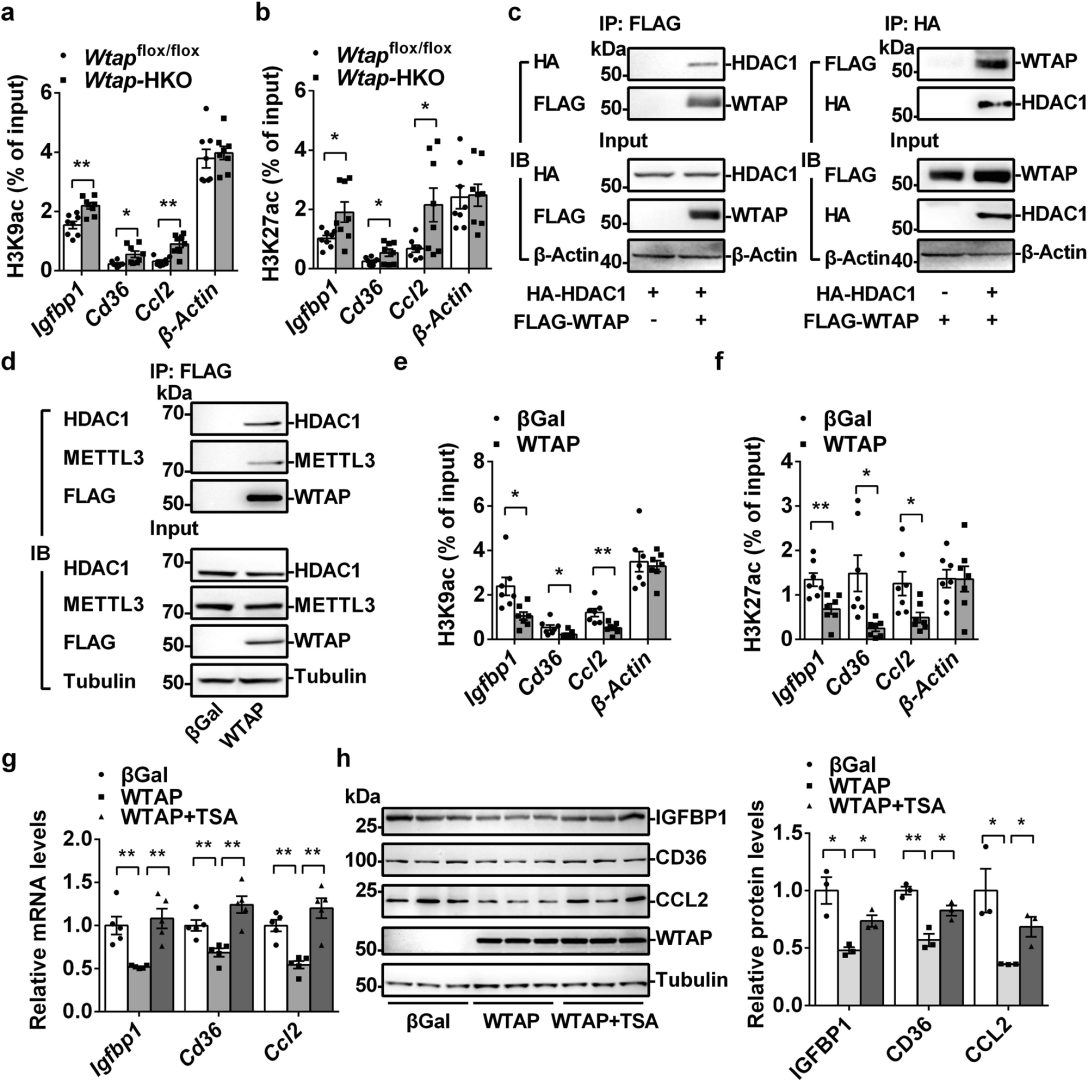
WTAP interacts with HDAC1 and regulates H3K9ac and H3K27ac levels in the promoters of *Igfbp1*, *Cd36* and *Ccl2* genes. **a**, **b** H3K9ac and H3K27ac levels in the promoters of *Igfbp1*, *Cd36* and *Ccl2* genes in the livers of *Wtap*^flox/flox^ and *Wtap-*HKO mice at 8 weeks old were measured by ChIP-qPCR (*n* = 8 for each group; H3K9ac: *Igfbp1*, *P* = 0.0013; *Cd36*, *P* = 0.0124; *Ccl2*, *P* = 0.00365; *β-Actin*, *P* = 0.6312; H3K27ac: *Igfbp1*, *P* = 0.0257; *Cd36*, *P* = 0.0462; *Ccl2*, *P* = 0.0233; *β-Actin*, *P* = 0.8985). **c** HA-HDAC1 expression vector was co-transfected with or without FLAG-WTAP expression vector in HEK293T cells. Total cell lysates were incubated with DNase1(200U/ml) at 37 °C for 30 min. These lysates were immunoprecipitated with FLAG or HA beads and then immublotted with anti-HA or anti-FLAG antibodies. **d** Primary hepatocytes were infected with Ad-βGal and Ad-FLAG-WTAP adenovirus. Total cell lysates were immunoprecipitated with FLAG beads and then immublotted with anti-HDAC1 antibodies. **e**, **f** H3K9ac and H3K27ac levels in the promoters of *Igfbp1*, *Cd36* and *Ccl2* genes in WTAP-overexpressing hepatocytes were measured by ChIP-qPCR (*n* = 7 for each group; H3K9ac: *Igfbp1*, *P* = 0.01095; *Cd36*, *P* = 0.0358; *Ccl2*, *P* = 0.005; *β-Actin*, *P* = 0.7053; H3K27ac: *Igfbp1*, *P* = 0.0049; *Cd36*, *P* = 0.0112; *Ccl2*, *P* = 0.0215; *β-Actin*, *P* = 0.9824). **g**, **h** Primary hepatocytes were infected with Ad-βGal and Ad-WTAP adenovirus, treated with or without TSA (2 μM) overnight, *Igfbp1*, *Cd36* and *Ccl2* mRNA levels were measured by qPCR (*n* = 5 for each group; *Igfbp1*: βGal versus WTAP *P* = 0.0018, WTAP versus WTAP+TSA, *P* = 0.0011; *Cd36*: βGal versus WTAP *P* = 0.00397, WTAP versus WTAP+TSA, *P* = 0.0009; *Ccl2*: βGal versus WTAP, *P* = 0.00045, WTAP versus WTAP+TSA, *P* = 0.000758). IGFBP1, CD36 and CCL2 protein levels were measured by immunoblotting (*n* = 3 for each group; IGFBP1: βGal versus WTAP *P* = 0.012, WTAP versus WTAP+TSA, *P* = 0.0113; CD36: βGal versus WTAP *P* = 0.0026, WTAP versus WTAP+TSA, *P* = 0.0226; CCL2: βGal versus WTAP *P*=0.0285, WTAP versus WTAP+TSA, *P* = 0.0193). n was the number of biologically independent mice or cell samples. The cell culture experiments were repeated for three times independently with similar results. Data represent the mean ± SEM. Significance was determined by unpaired two-tailed Student’s *t* test analysis. *p < 0.05. **p < 0.01. Source data are provided as a Source Data file.

Histone modifications have been shown to regulate chromatin accessibility and gene transcription. For example, the acetylation of H3K9 and H3K27 is associated with activation of gene transcription. To check which histone modification is involved in the activation of *Igfbp1*, *Cd36* and *Ccl2* transcription in the livers of *Wtap-*HKO mice, we performed ChIP assays. As shown in Fig. 6a, b, both H3K9ac and H3K27ac levels in the *Igfbp1*, *Cd36* and *Ccl2* promoters were significantly increased in the liver of *Wtap-*HKO mice, indicating that the increased acetylation of H3K9 and H3K27 contributes to the activation of *Igfbp1*, *Cd36* and *Ccl2* transcription. HDAC1 is known to repress the expression of target genes by deacetylating histone H3K9 and H3K27. We found that WTAP coimmunoprecipitated with HDAC1 in both HEK293T cells (Fig. 6c) and primary hepatocytes (Fig. 6d). In WTAP immunoprecipitated complex, METTL3 was also detected (Fig. 6d), which is consistent with previous reports^16^. As shown in Fig. 6e, f, both H3K9ac and H3K27ac levels in the *Igfbp1*, *Cd36* and *Ccl2* promoters were significantly decreased in the FLAG-WTAP-overexpressing hepatocytes, which indicates that WTAP was able to elicit deacetylation of H3K9 and H3K27 in the promoters of *Igfbp1*, *Cd36* and *Ccl2*. To confirm that the inhibition of *Igfbp1*, *Cd36* and *Ccl2* expression is mediated by WTAP-elicited histone deacetylation, primary hepatocytes were infected with Ad-FLAG-WTAP and treated with or without trichostatin A (TSA), a selective HDAC inhibitor. As shown in Fig. 6g, h, TSA reversed the suppression of *Igfbp1*, *Cd36* and *Ccl2* expression mediated by WTAP overexpression. These data demonstrate that WTAP regulates *Igfbp1*, *Cd36* and *Ccl2* transcription by modulating H3K9ac and H3K27ac in their promoters with the involvement of HDAC1.

### Nuclear WTAP is decreased in NASH

**Fig. 7:**
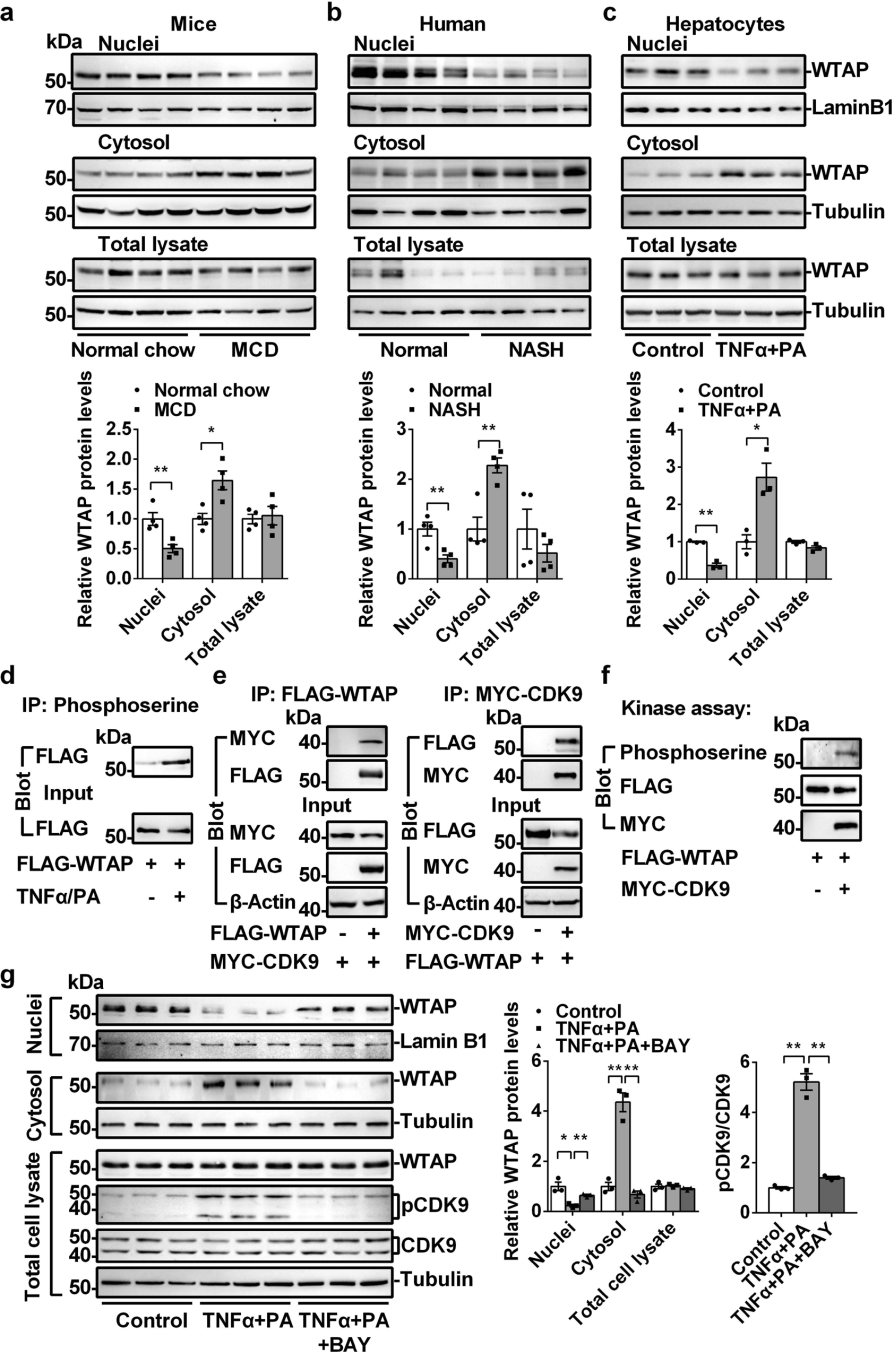
Nuclear WTAP is decreased in the NASH livers. **a** WTAP protein levels in nuclei, cytosol and total cell lysate from the livers of MCD (for 3 weeks)-fed mice were measured by immunoblotting (*n* = 4 for each group; Nuclei, *P* = 0.0081; Cytosol, *P* = 0.0123; Total cell lysate, *P* = 0.7573). **b** Immunoblotting of WTAP protein levels in nuclei, cytosol and total cell lysate from human NASH and normal liver tissues (*n* = 4 for each group; Nuclei, *P* = 0.0094; Cytosol, *P* = 0.0038; Total cell lysate, *P* = 0.3151). **c** Primary hepatocytes were isolated and treated with or without TNFα (100 ng/ml) and palmitate acid (PA) (1 mM) for 20 hours. WTAP protein levels in nuclei, cytosol, and total cell lysate were measured by immunoblotting (*n* = 3 for each group; Nuclei, *P* = 0.00046; Cytosol, *P* = 0.0146; Total cell lysate, *P* = 0.071). **d** Primary hepatocytes were infected with Ad-FLAG-WTAP adenovirus, and then treated with or without TNFα (100 ng/ml) and palmitate acid (PA) (1 mM) overnight. Total cell lysate was immunoprecipitated with anti-phosphoserine antibody and immunoblotted with anti-FLAG antibody. **e** MYC-CDK9 expression vector was co-transfected with or without FLAG-WTAP expression vector in HEK293T cells. Total cell lysates were immunoprecipitated with FLAG beads and then immublotted with anti-MYC or anti-FLAG antibodies. **f** In vitro kinase assay. This experiment was repeated for three times independently with similar results. **g** Primary hepatocytes were treated with vehicle, TNFα (100 ng/ml)/PA(1 mM), and TNFα (100 ng/ml)/PA(1 mM) plus BAY-1143572 (4 μM) for 20 hours. WTAP protein levels in nuclei, cytosol, and total cell lysate were measured by immunoblotting (*n* = 3 for each group; Control versus TNFα/PA: Nuclei *P* = 0.0114; Cytosol, *P* = 0.0012; Total cell lysate, *P* = 0.7439; TNFα/PA versus TNFα/PA+BAY-1143572: Nuclei *P* = 0.0032; Cytosol, *P* = 0.00079; Total cell lysate, *P* = 0.0595). CDK9, p-CDK9, Lamin B1, and Tubulin levels were also measured by immunoblotting (*n* = 3 for each group; Control versus TNFα/PA, *P* = 0.0002; TNFα/PA versus TNFα/PA+BAY-1143572, *P* = 0.0003). The samples were derived from the same experiment and the blots were processed in parallel. n was the number of biologically independent mice or cell samples. The cell culture experiments were repeated for three times independently with similar results. Data represent the mean ± SEM. Significance was determined by unpaired two-tailed Student’s *t* test analysis. *p < 0.05. **p < 0.01. Source data are provided as a Source Data file.

Hepatic deletion of *Wtap* results in NASH. We asked whether *Wtap* expression was regulated by NASH. As shown in Fig. 7a, b and Supplementary Fig. 17, total WTAP protein levels were not change in both mouse NASH model and human patients with NASH. However, nuclear WTAP protein levels were significantly decreased whereas cytosolic WTAP protein levels were increased in both mouse and human NASH samples (Fig. 7a, b and Supplementary Fig. 17). To further determine how WTAP was translocated from nucleus to cytosol in NASH, primary hepatocytes were isolated and treated with TNFα and palmitic acid (PA), which could mimic NASH in vitro^37,38^. As shown in Fig. 7c, TNFα and PA significantly decreased the nuclear WTAP protein levels and increased cytosolic WTAP protein levels. It has been shown that phosphorylation regulates nuclear/cytosolic localization^34,39^. TNFα and PA were able to induce phosphorylation of WTAP (Fig. 7d). It has been shown recently that CDK9 phosphorylated METTL3 and regulated its nuclear/cytosolic translocation in NASH livers^34^, which indicates that CDK9 may also regulate WTAP. To test this hypothesis, we performed Co-immunoprecipitation (Co-IP) and in vitro kinase assays. As shown in Fig. 7e, f, CDK9 interacted with WTAP, and CDK9 was able to phosphorylate WTAP. The p-CDK9 levels were elevated in NASH livers^34^, and TNFα/PA induced the phosphorylation of CDK9 in primary hepatocytes (Fig. 7g), which indicates that CDK9 may regulate the nuclear/cytosolic translocation of WTAP in NASH. Next, we tested whether inhibition of CDK9 can block the TNFα/PA-induced cytosolic translocation of WTAP. As shown in Fig. 7g, BAY-1143572, a CDK9 inhibitor, blocked TNFα/PA-induced p-CDK9 and the cytosolic translocation of WTAP. These data indicate that TNFα/PA/CDK9-mediated phosphorylation of WTAP may contribute to the reduction of nuclear WTAP levels in NASH livers, which may further contribute to the increased expression of CD36, IGFBP1 and CCL2, promoting NASH progression.

## Discussion

NASH is one of worldwide health problems, which is characterized by hepatic steatosis, liver injury, inflammation and fibrosis^40,41^. In NASH patients, the rate of lipolysis in WAT is increased and then FFAs are released, which contributes to steatosis (about 60% of hepatic triglycerides are derived from serum FFAs)^1^. Thus, it is important to clarify how liver dysfunction promotes lipolysis in WAT during NASH pathogenesis. In the present study, we show that nuclear WTAP is downregulated in NASH, and hepatic deletion of *Wtap* leads to NASH-like phenotypes due to increased lipolysis in eWAT, increased hepatic FFAs uptake and inflammation, which are strongly associated with increased expression of *Igfbp1*, *Cd36* and cytochemokines such as *Ccl2*, respectively. Mechanistically, WTAP directly binds to the promoters of *Igfbp1*, *Cd36* and *Ccl2* genes and recruits HDAC1, which causes deacetylation of H3K9 and H3K27, thus suppressing *Igfbp1*, *Cd36* and *Ccl2* transcription.

Hepatic deletion of *Wtap* leads to more pronounced steatosis, steatohepatitis and collagen deposition under the normal chow feeding condition. This is a very striking phenotype. *Wtap*-HKO mice show increased lipolysis in eWAT, which leads to higher serum FFAs levels, lower body weight and smaller eWAT. In human patients with NASH and mouse NASH model, the higher lipolysis rate in WAT has been reported^2,29^. These results indicate that liver dysfunction promotes lipolysis during NASH progression. We further show that liver-enriched secreted protein IGFBP1 is highly induced in *Wtap*-HKO mice. Serum IGFBP1 levels are also dramatically increased in *Wtap*-HKO mice as well as mouse NASH model. This observation is consistent with published human data that serum IGFBP1 is abnormally increased in NASH patients and serves as a marker for advanced fibrosis^30^. Recent study also shows that hepatic IGFBP1 is dramatically increased in NASH-derived HCC^42^. In other mouse model such as liver-specific carnitinbe palmitoyltransferase 2 (Cpt2) knockout mice, *Igfbp1*, *Fgf21* and *Gdf15* are induced under a ketogenic diet feeding condition, which likely leads to enhanced lipolysis in WAT, finally resulting in severe NASH with a complete absence of adipose triglyceride stores^43^. We provided direct evidence that IGFBP1 induces lipolysis in both white adipose tissue and primary adipocytes, which is likely due to the increased ADCY3/4/6/cAMP/PKA signaling pathway. Neutralization of IGFBP1 is able to decrease the higher lipolysis rate in eWAT of *Wtap*-HKO mice by inhibiting ADCY3/4/6/cAMP/PKA signaling pathway, which further ameliorates NASH progression in the *Wtap*-HKO mice. How IGFBP1 activates ADCY3/4/6/cAMP/PKA signaling pathway needs further study. It has been shown that IGFBP1 binds to intergrin β1 receptor via its RGD domain^44^. It would be interesting to test whether IGFBP1 binds to intergrin β1 receptor via its RGD domain and activates ADCY3/4/6/cAMP/PKA signaling pathway in white adipose tissue. In other diseases such as leukemia, IGFBP1 is also abnormally elevated, which contributes to increased lipolysis in WAT and systemic insulin resistance^45^. Neutralization of IGFBP1 also decreases lipolysis in WAT of leukemia mouse model^45^. Our results indicate that elevated IGFBP1 in NASH contributes to the increased rates of lipolysis in WAT, which further aggravates NASH progression.

In addition to elevated lipolysis and serum FFAs levels, hepatic deletion of *Wtap* increases FFAs uptake by increasing *Cd36* expression. Therefore, elevated serum FFAs could be rapidly translocated to the liver and reassembled into triglycerides, contributing to steatosis in *Wtap*-HKO mice. It has been well known that CD36 is abnormally upregulated in NAFLD and NASH^3^, and knockout of *Cd36* protects against MCD-induced NASH^46^. We noticed that factors related to lipogenesis, fatty acid oxidation and VLDL secretion were downregulated by hepatic deletion of *Wtap*, which indicates that lipogenesis less like contributes to steatosis in *Wtap*-HKO mice. Thus, the loss of *Wtap* in the liver promotes hepatic steatosis mainly by increasing both lipolysis in eWAT and FFAs uptake in the liver due to increased expression of *Igfbp1* and *Cd36*.

Besides liver steatosis, liver inflammation is also increased due to the inducation of cytochemokines, which further promotes liver injury and fibrosis in *Wtap*-HKO mice. Among the elevated cytochemokines, *Ccl2* and *Ccl22* are the top upregulated genes in *Wtap*-HKO livers and primary hepatocytes. It has been reported that CCL2 and its receptor CCR2 are strongly associated with NASH^11,12^, and inhibition of CCL2 and CCR2 is a therapeutic target for the treatment of NASH^47,48^. Therefore, abnormally elevated CCL2 expression contributes to NASH progression in *Wtap*-HKO mice. It should be noted that other upregulated cytochemokines such as *Tnfa*, *Il1β*, *Csf1*, *Ccl3*, *Ccl5*, *Ccl22*, *Cxcl2*, *Cxcl5* and *Cxcl10* may also contribute to NASH progression in *Wtap*-HKO mice.

Next, we try to address the question of how WTAP regulates *Igfbp1*, *Cd36* and cytochemokines (*Ccl2* as an example) expression. Although WTAP has been reported to regulate RNA splicing, RNA-seq analysis does not find alternative splicing of *Igfbp1*, *Cd36* and *Ccl2* mRNA. It has been reported that many RNA binding proteins can binding to DNA and regulate chromatin structure^35,36^. ATAC-seq peaks were significantly increased in the promoters of *Igfbp1*, *Cd36* and *Ccl2* in the livers of *Wtap-*HKO mice. Furthermore, WTAP binds to *Igfbp1*, *Cd36* and *Ccl2* promoters but does not bind to the *Actb* promoter in the FLAG-WTAP*-*overexpressing hepatocytes, as detected by ChIP-qPCR assays. Histone modifications have been shown to regulate chromatin accessibility and gene transcription. We checked which histone modification is involved in the activation of *Igfbp1*, *Cd36* and *Ccl2* transcription in the livers of *Wtap-*HKO mice by performing ChIP assays. Both H3K9ac and H3K27ac (active epigenetic marks) levels in the *Igfbp1*, *Cd36* and *Ccl2* promoters are significantly increased in the livers of *Wtap-*HKO mice, indicating that the increased acetylation of H3K9 and H3K27 contributes to the activation of *Igfbp1*, *Cd36* and *Ccl2* transcription. HDAC1 is known to repress the expression of target genes by deacetylating histone H3K9 and H3K27. We found that WTAP interacts with HDAC1 in the hepatocytes. Overexpression of *Wtap* decreases the levels of H3K9ac and H3K27ac in the promoters of *Igfbp1*, *Cd36* and *Ccl2*, leading to decreased transcription of *Igfbp1*, *Cd36* and *Ccl2*. Remarkably, inhibition of HDAC1 by TSA blocks the suppression of *Igfbp1*, *Cd36* and *Ccl2* transcription mediated by WTAP overexpression. These findings support a mechanism by which WTAP suppresses FFAs uptake and inflammation at least in part by recruiting HDAC1 to the promoters of *Igfbp1*, *Cd36* and *Ccl2*, where HDAC1 catalyzes the repressive deacetylation of H3K9 and H3K27. WTAP may also negatively regulate other cytochemokines such as *Tnfa*, *Il1β*, *Csf1*, *Ccl3*, *Ccl5*, *Ccl22*, *Cxcl2*, *Cxcl5* and *Cxcl10* by binding their promoters with the involvement of HDAC1. ChIP-seq analysis shows that the consensus of DNA binding motif of WTAP is TGASTCA, which is similar with binding motifs of Fosl2, Fra1/2, and Jun-AP1. Interestingly, ATAC-seq motif analysis shows that binding motifs of Fosl2, Fra1/2, and Jun-AP1 are differentially open in the livers of *Wtap*-HKO mice. It has been shown that overexpression of Fra-1 and Fra-2 blocks the progression of NAFL and NASH induced by a high fat diet (HFD) by suppressing transcription of *Pparg* through the action of inhibitory c-Jun/Fra-1 or c-Jun/Fra-2 heterodimers^49^, whereas elevated c-Jun promotes progression of NASH^50,51^. Because WTAP, Fosl2, Fra1/2 and Jun-AP1 share similar binding motifs, it is possible that they interact with each other, which further regulate gene expression and NASH progression. This possibility needs further investigation.

The nuclear WTAP protein levels are decreased in both human patients and mice with NASH, which indicates that decreased nuclear WTAP may contribute to the NASH progression. Decreased nuclear WTAP reduces HDAC1 activity in the promoters of *Igfbp1*, *Cd36* and *Ccl2*, which results in the higher levels of H3K9ac and H3K27ac in their promoters and further increases their transcription. This has been observed in *Wtap-*HKO mice. With respect to the cytosolic translocation of WTAP in NASH, (TNFα/PA)/CDK9-mediated serine phosphorylation of WTAP promotes cytosolic translocation of WTAP, while inhibition of CDK9 partially blocks cytosolic translocation of WTAP. In addition to WTAP, METTL3 is also regulated by CDK9 and translocated from nuclei to cytosol in NASH^34^. Hepatic deletion of *Mettl3* promotes diet-induced NASH by increasing the transcription of *Cd36* and *Ccl2*, which is due to increased chromatin accessibility in the promoter regions of *Cd36* and *Ccl2* with the involvement of HDAC1/2^34^. *Mettl3*-HKO mice do not show NASH phenotype under a normal chow feeding condition, which may be due to the normal IGFBP1 expression in *Mettl3*-HKO mice (GSE141325)^34^.

In addition to NASH, hepatic deletion of *Wtap* also impairs glucose homeostasis. Fasting and randomly feeding blood glucose levels are both reduced in *Wtap*-HKO mice. Hepatic gluconeogenesis is impaired, as revealed by decreased glucose levels after injection of glucose and pyruvate, respectively. Liver glycogen levels in *Wtap*-HKO mice are also decreased. The impaired glucose homeostasis observed in *Wtap*-HKO mice is likely due to the decreased expression of glucose metabolism-related genes such as *G6pase*, *Pepck*, *Glut2*, *Gys1*, *Pgc1α* and *Hnf4α*. ATAC-seq motif analysis shows that binding motifs of NHF4α are differentially close in the livers of *Wtap*-HKO mice, whereas they are differentially open in the livers of *Wtap*^flox/flox^ mice. How WTAP regulates the expression of *Hnf4α* and other glucose metabolism-related genes needs further study.

In conclusion, our data have demonstrated that hepatic WTAP is a key integrative regulator of ectopic lipid accumulation and inflammation during NASH progression with an involvement of IGFBP1, CD36 and cytochemokines such as CCL2. These data reveal a mechanism by which WTAP suppresses the transcription of *Igfbp1*, *Cd36* and *Ccl2 via* a histone modification pathway that includes the involvement of HDAC1. Decreased nuclear WTAP in NASH leads to the impairment of the WTAP/HDAC1 axis and increases the transcription of *Igfbp1*, *Cd36* and *Ccl2*, which further increases lipolysis in WAT, hepatic FFA uptake and liver inflammation, respectively, finally causing NASH pathogenesis. Our results also indicate that the WTAP/HDAC1 axis may serve as a drug target for the treatment of NASH.

## Method

### Animal experiments

Animal experiments were carried out in strict accordance with the Guide for the Care and Use of Laboratory Animals. Animal experiment protocols were approved by the Institutional Animal Care and Use Committee of Harbin Institute of Technology (HIT/IACUC). The approval number is IACUC-2018004. Mice were housed on a 12-h light/12-h dark cycle, temperature (24 ± 2 °C) and humidity (50% ± 10%) conditions. *Wtap*^flox/flox^ mice, in which the exon 4 of *Wtap* gene was flanked by two loxp sites, were generated using the CRISPR-Cas9 technique. Hepatocyte-specific *Wtap* knockout (*Wtap-*HKO) mice were generated by crossing *Wtap*^flox/flox^ mice with Alb-Cre mice. Both *Wtap*^flox/flox^ and Alb-Cre mice were in C57BL/6J background. Male mice were used for experiments. For GTTs and PTTs, *Wtap*^flox/flox^ and *Wtap-*HKO mice at 8 weeks old were fasted for 6 hours and intraperitoneally injected with glucose (2 g/kg body weight) or pyruvate (1 g/kg body weight). Blood glucose was measured at 0, 15, 30, 60 and 120 min after injection. For short time NASH diet feeding experiments, *Wtap*^flox/flox^ and *Wtap-*HKO mice at 6 weeks old were fed with a NASH diet (D18402002, Changzhou SYSE Bio-Tec. Co.,Ltd.) for five weeks. For inhibition of IGFBP1 in vivo, *Wtap-*HKO mice at 7 weeks old were treated with an anti-IGFBP1 (A11672, abclonal) antibody (4 μg antibody/mouse, i.v.) or equal amount of Rabbit IgG (bs-0295p, Bioss) for 5 days. Blood samples were collected from orbital sinus. The serum alanine aminotransferase (ALT) activities and FFA levels were measured with an ALT (C009-2-1, Nanjing Jiancheng) and FFA reagent set (A042-2-1, Nanjing Jiancheng), respectively^52,53^. Serum IGFBP1, TNFα, IL1β, CCL2 and IGF1 levels were measured using commercially available ELISA kits (Mouse IGFBP-1 ELISA Kit, EK0383, BOSTER; Mouse TNF-alpha ELISA Kit, KE10002, Proteintech; Mouse IL-1 beta ELISA Kit, KE10003, Proteintech; Mouse MCP-1 ELISA Kit, KE10006, Proteintech; Mouse/Rat IGF1 ELISA Kit, KE10032, Proteintech) following their manufacturer’s instructions, respectively. cAMP levels in eWAT were measured using cAMP ELISA kit (581001, Cayman). Liver glycogen levels were measured using Liver/Muscle glycogen assay kit (A043-1-1, Nanjing Jiancheng). Liver frozen sections were stained with Oil Red O (O0625, Sigma), Direct Red 80 (365548, Sigma), F4/80 (30325, CST, 1:200) and TUNEL Assay Kit (11684795910, Roche), respectively. Reagents used in this study were listed in the Supplementary Table 1.

### Human liver samples

Human liver samples were collected in the Third Affiliated Hospital of Sun Yat-sen University. This study was approved by the Research Ethics Committee of the Third Affiliated Hospital of Sun Yat-sen University, which complied with the Detailed Rules for the Implementation of the Regulations on the Management of Human Genetic Resources released by China’s Ministry of Science and Technology. Individual permission was obtained using standard informed consent procedures. The investigation conforms to the principles that are outlined in the Declaration of Helsinki regarding the use of human tissues. Detailed characteristics of patients with or without NASH have been shown in Supplementary Table 2.

### Free Fatty acid uptake assays

*Wtap*^flox/flox^ and *Wtap*-HKO mice were fasted overnight and then injected intraperitoneally with 20 μM BODIPY FL C_16_ for 20 min. Small pieces of liver samples were homogenized in a regular RIPA buffer (50mM Tris-HCL, 150 mM NaCl, 10 mM Na_4_P_2_O_7_, 2 mM EGTA, 1% NP40, 1 mM PMSF, 1 mM Na_3_VO_4_, pH7.5). Liver homogenates were mixed with 3 volumes of Dole’s reagent (heptane: 2-propanol: 2 N sulfuric acid; 10:40:1) and centrifuged at 18,000 g for 10 min. The fluorescence of BODIPY FL C_16_ in the top organic-phase supernatant were determined using a 488 nm excitation, 515 nm emission filter set (BioTek) and normalized to the protein concentration^34,54^.

For in vitro free fatty acid uptake assay, primary hepatocytes were isolated from *Wtap*^flox/flox^ and *Wtap-*HKO mice. Hepatocytes were incubated with BODIPY FL C_16_ (1 uM) for 30 min in serum free RPMI1640 medium, washed with PBS, and fixed with 4% PFA for 10 min at RT. The fluorescence of BODIPY FL C_16_ in hepatocytes was obtained using a fluorescent microscope (Olympus) and quantified using ImageJ version 1.39f (National Institute of Health)^34^.

### Primary hepatocyte culture and adenoviral infection

Primary hepatocytes were isolated from C57BL/6 control, *Wtap*^flox/flox^ and *Wtap-*HKO mice by liver perfusion with type II collagenase (Worthington Biochem, Lakewood, NJ) and cultured at 37 °C and 5% CO_2_ in RPMI1640 medium supplemented with 3% FBS^34^. Primary hepatocytes from C57BL/6 mice were infected with equal amount of βGal or FLAG-WTAP adenoviruses overnight, and then harvested in a regular RIPA buffer for immunoblotting or harvested in a TriPure Isolation Reagent (94015120, Roche, Mannheim, Germany) for RNA isolation.

### Real time quantitative PCR (RT-qPCR)

RT-qPCR was performed as shown previously^55^. Briefly, total RNAs were extracted using TriPure Isolation Reagent, and the first-strand cDNAs were synthesized using random primers and M-MLV reverse transcriptase (Promega, Madison, WI). RT-qPCR was done using Roche LightCycler 480 real-time PCR system (Roche, Mannheim, Germany). Real-time PCR quantification was analyzed using LightCycler 480 Software version 1.5.1. The expression of individual genes was normalized to the expression of 36B4. Primers for real time RT-qPCR were listed in Supplementary Table 3.

### Liver triacylglycerol (TAG) levels

Liver samples were homogenized in 1% acetic acid, and lipids were extracted using 80% chloroform/Methanol (2:1). The organic fractions were dried in chemical hood, resuspended in 3 M KOH, incubated at 70 °C for 1 h, mixed with MgCl_2_ (0.75 M), and centrifuged. The glycerol levels in the supernatant were measured using Free Glycerol Reagent (F6428, Sigma). Liver TAG levels were then calculated.

### Luciferase assays

The mouse *Igfbp1* promoter (from −1001 to −1), *Cd36* promoter (from −2001 to −1) and *Ccl2* promoter (from −2001 to −1) were cloned into pGL3 vectors, respectively. Transient transfection and luciferase assays were following previous publication^52,56^. Briefly, HEK293T cells were split in 24-well plate 16 h before transfection. *Igfbp1*, *Cd36* or *Ccl2* luciferase reporter plasmids and β-galactosidase (β-Gal) expressing plasmids were cotransfected with WTAP or empty expression vector by polyethylenimine (Sigma) into HEK293T cells for twenty-four hours. Empty plasmids were added to adjust the total amount of DNAs transfected. Cells were then lysed in reporter lysis buffer (Promega, Madison, WI), and luciferase activity was measured and normalized to β-Gal activity. Reagents used in this study were listed in the Supplementary Table 1.

### Transient transfection, immunoprecipitation and immunoblotting

HEK293T cells were split in 6-well plate 16 h before transfection. Indicated expression vectors (HA-HDAC1 or MYC-CDK9: 1 μg) were co-transfected with or without FLAG-WTAP expression vector (1 μg) in HEK293T cells for forty-eight hours. Cells were harvested in a regular RIPA buffer shown above. For the co-immunoprecipitation of HDAC1 and WTAP, total cell lysates were incubated with DNase1(200U/ml) at 37 °C for 30 min to digest genomic DNA. These lysates were then immunoprecipitated with FLAG, HA or MYC beads at 4 °C for 2 hours. Immunoblotting was performed using the indicated antibodies.

For immunoblotting, mouse liver tissues were homogenized in a regular RIPA buffer shown above. Human liver tissues were homogenized in another RIPA buffer (0.5% TritonX-100, 0.5% Sodium deoxycholate, 50 mM Tris-HCl, 2 mM EDTA, 150 mM NaCl, 0.1% SDS, 1 mM PMSF, 1 mM Na_3_VO_4_, pH7.5). Immunoblotting was performed using the indicated antibodies. Antibody dilutions were as follows: WTAP (10200-1-AP, Proteintech), 1:2500; METTL3 (96391, Cell Signaling Technology), 1:2500; LaminB1 (12987-1-AP, Proteintech), 1:5000; Tubulin (sc-5286, Santa cruz), 1:5000; β-Actin (60008-1-Ig, Proteintech), 1:5000; GAPDH (60004-1, Proteintech), 1:5000; CDK9 (11705-1-AP, Proteintech), 1:3000; pCDK9 (2549, Cell Signaling Technology), 1:3000; CD36 (18836-1-AP, Proteintech), 1:5000; CCL2 (66272-1-Ig, Proteintech), 1:2500; FLAG (F1804, Sigma), 1:5000; Phosphoserine (AB1603, Sigma), 1:1000; MYC (16286-1-AP, Proteintech), 1:5000; ATGL (55190-1-AP, Proteintech), 1:5000; HDAC1 (5356, Cell Signaling Technology), 1:2500; ADCY3 (bs-20272R, Bioss), 1:3000; ADCY4 (bs-3921R, Bioss), 1:3000; ADCY6 (14616-1-AP, Proteintech); IGFBP1 (A11672, ABclonal), 1:3000; HSL (A15686, ABclonal), 1:5000; phospho-HSL-S563 (AP0851, ABclonal), 1:5000; Phospho-(Ser/Thr) PKA Substrate (9621, Cell Signaling Technology), 1:3000; and Cleaved caspase 3 (9661, Cell Signaling Technology), 1:2500. Antibodies were also listed in the Supplementary Table 1 and Reporting Summary.

### In vitro kinase assay

In vitro kinase assay was shown previously^34^. Briefly, MYC-CDK9 was immunopurified using an anti-MYC magnetic Beads (B26301, Bimake). MYC-CDK9 was incubated with purified FLAG-WTAP (0.87 μg) in kinase buffer (20 mM HEPES, pH 7.5–7.6, 33 μM ATP, 10 mM MgCl_2_, 50 mM NaCl, 1 mM PMSF and Phosphatase Inhibitor Cocktail 1) at 30 °C for 1 hour. FLAG-WTAP (0.87 μg) in the kinase buffer without MYC-CDK9 was served as a control. The reactions were stopped by adding SDS-PAGE loading buffer and boiling for 5 min. Proteins were immunoblotted with the antibodies against phosphoserine, FLAG and MYC, respectively. Antibody dilutions were as follows: phosphoserine (AB1603, Sigma), 1:1000; FLAG (F1804, Sigma), 1:5000; and MYC (16286-1-AP, Proteintech), 1:5000.

### Chromatin immunoprecipitation (ChIP) and ChIP-sequencing assays

The livers were cutted into 1–2 mm^3^ pieces that were fixed with 1% formaldehyde (freshly made) for 10 min. Primary hepatocytes were also fixed with 1% formaldehyde for 10 min. The nuclei were isolated from livers or primary hepatocytes and subjected to sonication (M220, Focused-ultrasonicator, Covaris) to break genomic DNA into 500- to 1000-bp fragments using truChIP Chromatin Shearing Kit (520127, Covaris). The samples were precleared with salmon sperm DNA (2 mg/mL) and protein A agarose beads. Then, the samples were immunoprecipitated with 30 μl Anti-FLAG M2 Magnetic Beads (M8823, Millipore) or 3 μL antibodies against H3K9ac (PTM-122, Jingjie PTM BioLab) or H3K27ac (4353, Cell Signaling Technology) at 4 °C for 3 h. For H3K9ac- or H3K27ac- ChIP, 45 μL Protein A Agarose Beads (20333, Pierce) were added and incubated at 4 °C for 2 h. Precipitates were washed sequentially with buffer 1 (0.1% SDS, 1% Triton X-100, 2 mM EDTA, 150 mM NaCl, and 20 mM Tris-HCl [pH 8.1]), buffer 2 (0.1% SDS, 1% Triton X-100, 2 mM EDTA, 500 mM NaCl, and 20 mM Tris-HCl [pH 8.1]), buffer 3 (0.25 M LiCl, 1% Nonidet P-40, 1% deoxycholate, 1 mM EDTA, 150 mM NaCl, and 10 mM Tris-HCl [pH 8.1]), and buffer 4 (10 mM Tris-HCl and 1 mM EDTA [pH 8.1]). DNA was eluted with 100 μL of elution buffer (1% SDS and 0.1 M NaHCO_3_) at 65 °C for 30 minutes and 2 μL Proteinase K (P1120, Solarbio) was addedand incubated at 55 °C overnight to reverse protein-DNA crosslinking. DNA was extracted with DNA Purification Kit (D1300, Solarbio) and used for qPCR and ChIP-seq analysis. Primers for ChIP-qPCR were listed in Supplementary Table 3. Reagents and antibodies used in this study were listed in the Supplementary Table 1.

For ChIP-seq, the purified DNA was used for library preparation. Library quality was assessed on the Agilent Bioanalyzer 2100 system. Pair-end sequencing of sample was performed on Illumina platform (Illumina, CA, USA). Raw data (raw reads) of fastq format were processed using fastp (version 0.19.11) software. In this step, clean data (clean reads) were obtained by removing reads containing adapter, reads containing ploy-N and low quality reads from raw data. At the same time, Q20, Q30 and GC content of the clean data were calculated. All the downstream analyses were based on the clean data with high quality. For a specific ChIP-seq binding site, individual reads mapping to the plus or minus strand and present significant enrich. MACS2 scan all the genome with a specific window size and calculate the reads enrichment level. A particular number of windows were used as samples to build enrichment model and to predict fragment size. The following peak calling analysis was based on this predicted fragment size. After mapping reads to the reference genome, we used the MACS2 (version 2.1.0) peak calling software to identify regions of IP enrichment over background. A q-value threshold of 0.05 was used for all data sets. After peak calling, the distribution of chromosome distribution, peak width, fold enrichment, significant level and peak summit number per peak were all displayed. Homer was used to detect the denovo sequence motif and the matched known motifs. FLAG-WTAP-ChIP-seq data support the findings of this study have been deposited in GEO under accession code GSE198023.

### Nuclear extract preparation

Liver tissues were homogenized in a lysis buffer (20 mM HEPES, 1 mM EDTA, 250 mM sucrose, 1 mM PMSF, 1 mM Na_3_VO_4_ and 0.5 mM DTT, pH 7.4). Primary hepatocytes were harvested in the above lysis buffer. Tissue or cell lysates were centrifuged sequentially at 1100 g and 4000 g at 4 °C. Nuclear protein was extracted from the pellets using a RIPA buffer (0.5% TritonX-100, 0.5% Sodium deoxycholate, 50 mM Tris-HCl, 2 mM EDTA, 150 mM NaCl, 0.1% SDS, 1 mM PMSF, 1 mM Na_3_VO_4_, pH7.5).

### LC-MS/MS analysis of serum free fatty acids

*Wtap*^flox/flox^ and *Wtap-*HKO mice at 8 weeks old were fasted for six hours. Blood samples were collected from orbital sinus. Palmitic acid along with other free fatty acids were extractedfrom serum samples using a solid phase extraction (SPE) method. Briefly, 1450 µL of cold methanol: water (30:70, v/v) containing 0.1% acetic acid and 0.002% BHT was used to extract free fatty acids from 50 µL serum. Serum was centrifuged at 10,000 × *g* for 15 min at 4 °C,and collected the supernatant in 1.5 mL centrifuge tubes. Thesupernatant was applied to SPE cartridge that had been preconditioned with 3 mL of methanol in 0.1% BHT and 0.1% acetic acid followed by 3 mL of water. Then, cartridge was washed with 5 mL cold water and 5 mL cold methanol: water (20:75, v/v) in succession. After sorbent dryness, extracts were eluted with 2 mL of cold ethyl acetate. The eluate was evaporated under nitrogen and stored at −80 °C prior to LC-MS/MS analysis.

For LC-MS/MS analysis, samples were resuspended in 30 µL of methanol and 10 µL was injected into 5500 QTRAP triple-quadrupole mass spectrometer (SCIEX) coupled to HPLC system (Shimadzu). Extracts were eluted by using a C30 column (3 μm, 2.1 × 150 mm, Acclaim) with a flow rate of 260 µL/min using buffer A (10 mM ammonium formate, at a 60:40 ratio with acetonitrile: water), adjusted with ammonium hydroxide, and buffer B (10 mM ammonium formate, at a 90:10 ratio with isopropanol: acetonitrile). Gradients: 32% B was held for 1.5 min at the initial; 45% B at 4 min; 52% B at 5 min; 58% B at 8 min; 66% B at 11 min; 70% B at 14 min; 75% B at 18 min; 97% B at 21 min; 97% B at 25 min; 32% B at 25.01 min; 32% B at 33 min. All the ions were acquired by multiple reaction monitoring (MRM) transitions in negative mode (Supplementary Table 4). ESI voltage was −4500 V,curtain gas was 25, collision gas was 10, source temperature was 475 °C.

For quantification of serum palmitic acid levels, linearity of the method was evaluated by analyzing standard solutions at distinct concentration levels. The calibration curve was constructed by plotting the peak area of standard analytes concentrations using a 1/x2 weighted linear regression model. This experiment was performed at five concentrations: 10 μM, 30 µM, 50 μM, 100 μM, and 300 μM. (Supplementary Fig. 18).

### m^6^ARIP-sequencing

m^6^ARIP-seq assay was performed using a standard protocol with some modifications^33,34,57^. Total RNA was extracted using Tripure Isolation Reagent (94015120, Roche, Mannheim, Germany) from livers of *Wtap*^flox/flox^ and *Wtap*-HKO mice at 8 weeks old. Each sample (300 μg total RNA) was pooled from 5 mice for each group. Two independent biological replicates for each group were used for m^6^ARIP-seq. mRNA was isolated using Dynabeads™ mRNA Purification Kit (Invitrogen) following the manufacturer’s instructions. Fragmented mRNA was incubated with the m^6^A antibody (202003, Synaptic System) for immunoprecipitation. Immunoprecipitated mRNAs or Input was then used for library construction with NEBNext ultra RNA library prepare kit (New England Biolabs). The library preparations were sequenced on an Illumina Novaseq 6000 platform with a paired-end read length of 150 bp according to the standard protocols. Index of the reference genome was built using BWA v0.7.12 and clean reads were aligned to the reference genome (Ensemble_GRCm38.92) using BWA mem v 0.7.12. The m^6^A peaks were detected by exomePeak R package (version 2.16.0). The motif search was detected by HOMER (version 4.9.1). Differential peak calling was performed using exomePeak R package (version 2.16.0) with parameters of *P*-value less than 0.01 and fold change more than 1. Using the same method, genes associated with different peaks were identified and also do GO and KEGG enrichment analysis. m^6^ARIP-seq data support the findings of this study have been deposited in GEO under accession code GSE192884.

### ATAC-sequencing and RNA-sequencing

Assay for transposase-accessible chromatin-sequencing (ATAC-seq) was performed using a standard method with some modifications^34,58,59^. Each liver sample was pooled from three *Wtap*^flox/flox^ and *Wtap-*HKO mice at 8 weeks old, respectively. Three independent biological replicates of each group were used for ATAC-seq. Nuclei were extracted from liver samples of *Wtap*^flox/flox^ and *Wtap-*HKO mice. The nuclei pellet were resuspended in the Tn5 transposase reaction mix and then incubated at 37 °C for 30 min. After transposition, Equimolar Adapter1 and Adatper 2 were added. PCR was then performed to amplify the library. After the PCR reaction, libraries were purified with the AMPure beads, and library quality was assessed with Qubit. The clustering of the index-coded samples was performed on a cBot Cluster Generation System using TruSeq PE Cluster Kit v3-cBot-HS (Illumina) according to the manufactuer’s instructions. After cluster generation, the library preparations were sequenced on an Illumina NovaSeq 6000 platform and 150 bp paired-end reads were generated. ATAC-seq analysis was performed using a standard protocol^34,58,59^. ATAC-seq data have been deposited in GEO under accession code GSE168945. RNA-seq analysis was performed in the livers of *Wtap*^flox/flox^ and *Wtap-*HKO mice at 8 weeks old. Three independent biological mice of each group were used for RNA-seq, which was performed as described previously^33,56,57,60^. Briefly, mRNA profiles were generated by deep sequencing using an Illumina Novaseq 6000 platform. Paired-end clean reads were aligned to the mouse reference genome (Ensemble_GRCm38.p6) with TopHat (version 2.0.12), and the aligned reads were used to quantify mRNA expression by using HTSeq-count (version 0.6.1). Alternative splicing was analyzed by rMATS software (version 3.2.5). False Discovery Rate (FDR) <0.05 was considered statistically significant. RNA-seq data have been deposited in GEO under accession code GSE168850.

### Statistical analysis

Data were analyzed using GraphPad Prism 6.02. Data were presented as means ± SEM. Differences between groups were analyzed by two-tailed Student’s *t* tests. *p* < 0.05 was considered statistically significant. *p < 0.05; **p < 0.01.

### Reporting summary

Further information on research design is available in the Nature Research Reporting Summary linked to this article.

## Supplementary information

Supplementary Information

Reporting Summary

Description of Additional Supplementary Files

Supplementary Data 1

Supplementary Data 2

Supplementary Data 3

Supplementary Data 4

Supplementary Data 5

## Data Availability

The RNA-seq data generated in this study have been deposited in the GEO database under accession code GSE168850. The m^6^ARIP-seq data generated in this study have been deposited in the GEO database under accession code GSE192884. The ATAC-seq data generated in this study have been deposited in the GEO database under accession code GSE168945. The ChIP-seq data generated in this study have been deposited in the GEO database under accession code GSE198023. The source data underlying Fig. 1a–m, 2b–h, 3a–i, 4a–p, 5c, f–h, 6a–h, and 7a–g and Supplementary Figs. 1b–e, 2a–l, 3a–f, 4a, b, 5a–e, 6a, b, 7a, b, 8a–c, 9a, b, 10a–c, 17 and 18 are provided as a Source Data file. All other data generated or analysed during this study are included in this published article (and its Supplementary Information files).

## Source data

Source Data

## References

[1] Donnelly KL (2005). Sources of fatty acids stored in liver and secreted via lipoproteins in patients with nonalcoholic fatty liver disease. J. Clin. Invest..

[2] Armstrong MJ (2014). Abdominal subcutaneous adipose tissue insulin resistance and lipolysis in patients with non-alcoholic steatohepatitis. Diabetes Obes. Metab..

[3] Miquilena-Colina ME (2011). Hepatic fatty acid translocase CD36 upregulation is associated with insulin resistance, hyperinsulinaemia and increased steatosis in non-alcoholic steatohepatitis and chronic hepatitis C. Gut.

[4] Kahn CR, Wang G, Lee KY (2019). Altered adipose tissue and adipocyte function in the pathogenesis of metabolic syndrome. J. Clin. Invest..

[5] Polyzos SA, Perakakis N, Mantzoros CS (2019). Fatty liver in lipodystrophy: a review with a focus on therapeutic perspectives of adiponectin and/or leptin replacement. Metab.: Clin. Exp..

[6] Liu L (2014). Adipose-specific knockout of SEIPIN/BSCL2 results in progressive lipodystrophy. Diabetes.

[7] Softic S (2016). Lipodystrophy due to adipose tissue-specific insulin receptor knockout results in progressive NAFLD. Diabetes.

[8] Agarwal AK (2002). AGPAT2 is mutated in congenital generalized lipodystrophy linked to chromosome 9q34. Nat. Genet..

[9] Savage DB (2009). Mouse models of inherited lipodystrophy. Dis. Models Mechanisms.

[10] Rubio-Cabezas O (2009). Partial lipodystrophy and insulin resistant diabetes in a patient with a homozygous nonsense mutation in CIDEC. EMBO Mol. Med..

[11] Haukeland JW (2006). Systemic inflammation in nonalcoholic fatty liver disease is characterized by elevated levels of CCL2. J. Hepatol..

[12] Miura K, Yang L, Rooijen NV, Ohnishi H, Seki E (2012). Hepatic recruitment of macrophages promotes nonalcoholic steatohepatitis through CCR2. Am. J. Physiol.-Gastrointest. Liver Physiol..

[13] Little NA, Hastie ND, Davies RC (2000). Identification of WTAP, a novel Wilms’ tumor 1-associating protein. Hum. Mol. Genet..

[14] Scholler E (2018). Interactions, localization, and phosphorylation of the m(6)A generating METTL3-METTL14-WTAP complex. RNA.

[15] Liu J (2014). A METTL3-METTL14 complex mediates mammalian nuclear RNA N6-adenosine methylation. Nat. Chem. Biol..

[16] Ping XL (2014). Mammalian WTAP is a regulatory subunit of the RNA N6-methyladenosine methyltransferase. Cell Res..

[17] Moindrot B (2015). A Pooled shRNA Screen Identifies Rbm15, Spen, and Wtap as Factors Required for Xist RNA-Mediated Silencing. Cell Rep..

[18] Horiuchi K (2006). Wilms’ tumor 1-associating protein regulates G2/M transition through stabilization of cyclin A2 mRNA. Proc. Natl Acad. Sci. USA.

[19] Kobayashi, M. et al. The RNA Methyltransferase Complex of WTAP, METTL3, and METTL14 Regulates Mitotic Clonal Expansion in Adipogenesis. *Mol. Cell Biol.* **38**, 10.1128/MCB.00116-18 (2018). 10.1128/MCB.00116-18 PMC6066751 29866655

[20] Xie, W. et al. Physiological functions of Wilms’ tumor 1-associating protein and its role in tumourigenesis. *J. Cell Biochem*., 10.1002/jcb.28402 (2019). 10.1002/jcb.28402 30756410

[21] Bansal H (2014). WTAP is a novel oncogenic protein in acute myeloid leukemia. Leukemia.

[22] Fukusumi Y, Naruse C, Asano M (2008). Wtap is required for differentiation of endoderm and mesoderm in the mouse embryo. Dev. Dyn..

[23] Friedman SL, Neuschwander-Tetri BA, Rinella M, Sanyal AJ (2018). Mechanisms of NAFLD development and therapeutic strategies. Nat. Med..

[24] Kawano Y, Cohen DE (2013). Mechanisms of hepatic triglyceride accumulation in non-alcoholic fatty liver disease. J. Gastroenterol..

[25] Tilg H (1992). Serum levels of cytokines in chronic liver diseases. Gastroenterology.

[26] Simpson KJ, Lukacs NW, Colletti L, Strieter RM, Kunkel SL (1997). Cytokines and the liver. J. Hepatol..

[27] Meinken, J., Walker, G., Cooper, C. R. & Min, X. J. MetazSecKB: the human and animal secretome and subcellular proteome knowledgebase. *Database* **2015**, 10.1093/database/bav077 (2015). 10.1093/database/bav077 PMC4529745 26255309

[28] Uhlén M (2015). Proteomics. Tissue-based map of the human proteome. Science.

[29] Tanaka N (2014). Role of white adipose lipolysis in the development of NASH induced by methionine- and choline-deficient diet. Biochim. Biophys. Acta.

[30] Hagström H, Stål P, Hultcrantz R, Brismar K, Ansurudeen I (2017). IGFBP-1 and IGF-I as markers for advanced fibrosis in NAFLD - a pilot study. Scand. J. Gastroenterol..

[31] Lewitt MS, Dent MS, Hall K (2014). The Insulin-Like Growth Factor System in Obesity, Insulin Resistance and Type 2 Diabetes Mellitus. J. Clin. Med..

[32] Haluzik M (2003). Insulin resistance in the liver-specific IGF-1 gene-deleted mouse is abrogated by deletion of the acid-labile subunit of the IGF-binding protein-3 complex: relative roles of growth hormone and IGF-1 in insulin resistance. Diabetes.

[33] Wang Y (2020). METTL3 is essential for postnatal development of brown adipose tissue and energy expenditure in mice. Nat. Commun..

[34] Li X (2021). The methyltransferase METTL3 negatively regulates nonalcoholic steatohepatitis (NASH) progression. Nat. Commun..

[35] Van Nostrand EL (2020). A large-scale binding and functional map of human RNA-binding proteins. Nature.

[36] Xiao R (2019). Pervasive Chromatin-RNA Binding Protein Interactions Enable RNA-Based Regulation of Transcription. Cell.

[37] Ricchi M (2009). Differential effect of oleic and palmitic acid on lipid accumulation and apoptosis in cultured hepatocytes. J. Gastroenterol. Hepatol..

[38] Kudo H (2009). Lipopolysaccharide triggered TNF-α-induced hepatocyte apoptosis in a murine non-alcoholic steatohepatitis model. J. Hepatol..

[39] Bauer NC, Doetsch PW, Corbett AH (2015). Mechanisms regulating protein localization. Traffic.

[40] Diehl AM, Day C (2017). Cause, pathogenesis, and treatment of nonalcoholic steatohepatitis. N. Engl. J. Med..

[41] Machado MV, Diehl AM (2016). Pathogenesis of nonalcoholic steatohepatitis. Gastroenterology.

[42] Dechassa ML (2018). Identification of chromatin-accessible domains in non-alcoholic steatohepatitis-derived hepatocellular carcinoma. Mol. Carcinogenesis.

[43] Lee J, Choi J, Scafidi S, Wolfgang MJ (2016). Hepatic Fatty acid oxidation restrains systemic catabolism during starvation. Cell Rep..

[44] Wang X, Wei W, Krzeszinski JY, Wang Y, Wan Y (2015). A liver-bone endocrine relay by IGFBP1 promotes osteoclastogenesis and mediates FGF21-induced bone resorption. Cell Metab..

[45] Ye H (2018). Subversion of systemic glucose metabolism as a mechanism to support the growth of leukemia cells. Cancer Cell.

[46] Bieghs V (2010). Role of scavenger receptor A and CD36 in diet-induced nonalcoholic steatohepatitis in hyperlipidemic mice. Gastroenterology.

[47] Baeck C (2012). Pharmacological inhibition of the chemokine CCL2 (MCP-1) diminishes liver macrophage infiltration and steatohepatitis in chronic hepatic injury. Gut.

[48] Lefebvre E (2016). Antifibrotic effects of the dual CCR2/CCR5 antagonist cenicriviroc in animal models of liver and kidney fibrosis. PLOS ONE.

[49] Hasenfuss SC (2014). Regulation of steatohepatitis and PPARγ signaling by distinct AP-1 dimers. Cell Metab..

[50] Schulien I (2019). The transcription factor c-Jun/AP-1 promotes liver fibrosis during non-alcoholic steatohepatitis by regulating Osteopontin expression. Cell Death Differ..

[51] Dorn C (2014). Increased expression of c-Jun in nonalcoholic fatty liver disease. Lab. Investig..

[52] Ren X (2017). A small-molecule inhibitor of NF-κB-inducing kinase (NIK) protects liver from toxin-induced inflammation, oxidative stress, and injury. FASEB J..

[53] Ding K (2022). GBP5 promotes liver injury and inflammation by inducing hepatocyte apoptosis. FASEB J..

[54] Demers A (2015). PCSK9 induces CD36 degradation and affects long-chain Fatty Acid uptake and triglyceride metabolism in adipocytes and in mouse liver. Arteriosclerosis, Thrombosis, Vasc. Biol..

[55] Li X (2018). Islet α-cell Inflammation Induced By NF-κB inducing kinase (NIK) Leads to Hypoglycemia, Pancreatitis, Growth Retardation, and Postnatal Death in Mice. Theranostics.

[56] Jia L, Jiang Y, Li X, Chen Z (2020). Purbeta promotes hepatic glucose production by increasing Adcy6 transcription. Mol. Metab..

[57] Li X, Jiang Y, Sun X, Wu Y, Chen Z (2021). METTL3 is required for maintaining β-cell function. Metab.: Clin. Exp..

[58] Buenrostro JD, Giresi PG, Zaba LC, Chang HY, Greenleaf WJ (2013). Transposition of native chromatin for fast and sensitive epigenomic profiling of open chromatin, DNA-binding proteins and nucleosome position. Nat. Methods.

[59] Corces MR (2017). An improved ATAC-seq protocol reduces background and enables interrogation of frozen tissues. Nat. Methods.

[60] Li X (2020). Activation of NF-κB-inducing kinase in Islet β cells causes β cell failure and diabetes. Mol. Ther.: J. Am. Soc. Gene Ther..

