# Supplementary material for: Deficiency of WTAP in hepatocytes induces lipoatrophy and non-alcoholic steatohepatitis (NASH)
Source: Nat Commun. 2022 Aug 4;13:4549. doi: 10.1038/s41467-022-32163-w (PMC9352699; doi:10.1038/s41467-022-32163-w)
Supplement: Supplementary file 1 — Supplementary Information [file 41467_2022_32163_MOESM1_ESM.docx]

Deficiency of WTAP in hepatocytes induces lipoatrophy and non-alcoholic steatohepatitis (NASH)

Xinzhi Li, Kaixin Ding, Xueying Li, Bingchuan Yuan, Yuqin Wang, Zhicheng Yao, Shuaikang Wang, He Huang, Bolin Xu, Liwei Xie, Tuo Deng, Xiao-wei Chen, and Zheng Chen

**Supplementary Figure 1. Generation of *Wtap*^flox/flox^ and *Wtap-*HKO** **mice**

(a) Generation of *Wtap*^flox/flox^ and *Wtap-*HKO mice.

(b) Genotyping of WT, *Wtap*^flox/-^ (HE) and *Wtap*^flox/flox^ (HO) mice. This experiment was repeated at least three times independently with same results.

(c-e) The body weight, liver weight and liver TAG levels were measured in 8-week-old *Wtap*^flox/flox^ and *Alb*-Cre mice fed a normal chow diet (n=5 for each group)**.** n was the number of biologically independent mice. Data represent the mean ± SEM. Source data are provided as a Source Data file.

**Supplementary Figure 2. Hepatic deletion of *Wtap* promotes diet-induced NASH**

*Wtap*^flox/flox^ and *Wtap-*HKO mice were fed with NASH diet for five weeks.

(a) Representative pictures of livers from *Wtap*^flox/flox^ and *Wtap-*HKO mice.

(b) Liver weights (n=8 for each group; *P*<0.0001).

(c) Liver TAG levels (n=8 for each group; *P*=0.00075).

(d) Oil Red O staining was performed in the liver sections from *Wtap*^flox/flox^ and *Wtap-*HKO mice (n=5 for each group). n was the number of biologically independent mice. Mice of the same genotype showed similar phenotypes. Representative Oil Red O staining images were shown.

(e) Serum ALT activity in *Wtap*^flox/flox^ and *Wtap-*HKO mice (n=8 for each group; *P*<0.0001).

(f) The TUNEL-positive cells in liver sections of *Wtap*^flox/flox^ and *Wtap-*HKO mice (n=5 for each group; *P*<0.0001).

(g) Cleaved caspase 3 levels were measured by immunoblotting (n=4). The samples were derived from the same experiment and the blots were processed in parallel.

(h) F4/80 immunostaining of liver sections (n=5 for each group; *P*<0.0001).

(i) Sirius Red staining of liver sections (n=5 for each group; *P*<0.0001).

(j) RT-qPCR analysis of fibrosis-related mRNA levels (n=8 for each group; *Collagen IA1*, *P*<0.0001; *αSma*, *P*<0.0001; *Mmp9*, *P*=0.0005; *Tgfb1*, *P*=0.00019).

(k) RT-qPCR analysis of cytochemokine mRNA levels (n=8 for each group; *Tnfα*, *P*<0.0001; *Il1β*, *P*=0.0155; *Il6*, *P*=0.0012; *iNos*, *P*<0.0001; *Infg*, *P*=0.0036; *Cd14*, *P*<0.0001; *Csf1*, *P*<0.0001; *Ccl2*, *P*<0.0001; *Ccl3*, *P*<0.0001; *Ccl5*, *P*<0.0001; *Ccl22*, *P*<0.0001; *Ccr2*, *P*<0.0001; *Cxcl2*, *P*=0.0073; *Cxcl5*, *P*<0.0001; *Cxcl10*, *P*<0.0001; *Cx3cl1*, *P*<0.0001).

(l) Serum cytokine levels (n=8 for each group; TNFα, *P*=0.00016; CCL2, *P*<0.0001; IL1β, *P*=0.0005).

n was the number of biologically independent mice. Data represent the mean ± SEM. Significance was determined by unpaired two-tailed Student's *t* test analysis. *, *p*< 0.05. **, *p*< 0.01. Source data are provided as a Source Data file.

**Supplementary Figure 3. *Wtap*-HKO mice show impaired hepatic glucose homeostasis**

(a) *Wtap*^flox/flox^ and *Wtap-*HKO mice were fasted for 6 hours and fasting blood glucose levels were measured (n=9 for each group; *P*=0.0044).

(b) Randomly feeding blood glucose levels were measured in *Wtap*^flox/flox^ and *Wtap-*HKO mice at 8:00-9:00 AM (n=9 for each group; *P*=0.00037).

(c) *Wtap*^flox/flox^ and *Wtap-*HKO mice were fasted for 6 hours and GTTs were measured (n=7 for each group; 0, *P*<0.0001; 15, *P*=0.000285; 30, *P*=0.0001; 60, *P*=0.0007; 120, *P*=0.00026)**.**

(d) *Wtap*^flox/flox^ and *Wtap-*HKO mice were fasted for 6 hours and PTTs were measured (n=7 for each group; 0, *P*=0.00676; 15, *P*<0.0001; 30, *P*=0.0004; 60, *P*=0.0002; 120, *P*=0.000578)**.**

(e) Relative mRNA levels (*Wtap*^flox/flox^, n=10; *Wtap*-HKO, n=8; *G6pase*, *P*=0.0035; *Pepck*, *P*=0.0425; *Glut2*, *P*=0.0036; *Gys1*, *P*=0.016; *Pgc1α*, *P*=0.0003; *Hnf4α*, *P*<0.0001)**.**

(f) Liver glycogen levels (n=8 for each group; *P*<0.0001)**.**

n was the number of biologically independent mice. Data represent the mean ± SEM. Significance was determined by unpaired two-tailed Student's *t* test analysis. *, *p*< 0.05. **, *p*< 0.01. Source data are provided as a Source Data file.

**Supplementary Figure 4. Hepatic deletion of *Wtap* does not affect lipolysis in iWAT.**

(a)The relative iWAT weights in *Wtap*^flox/flox^ and *Wtap-*HKO mice at 8 weeks old (n=8 for each group).

(b) p-HSL, HSL, p-PKA substrate, ATGL, IGFBP1 and GAPDH protein levels were measured by immunoblotting in the eWAT of *Wtap*^flox/flox^ and *Wtap-*HKO mice (n=4 for each group). The liver sample from WT mouse serves as a positive control for IGFBP1. The samples were derived from the same experiment and the blots were processed in parallel.

Data represent the mean ± SEM. n was the number of biologically independent mice. Source data are provided as a Source Data file.

**Supplementary Figure 5.** ***Wtap-*HKO mice display increased lipolysis in the eWAT.**

*Wtap*^flox/flox^ and *Wtap-*HKO mice were fed with NASH diet for five weeks.

(a)Serum FFA levels (n=8 for each group; *P*=0.0006).

(b) The relative eWAT, iWAT and iBAT weight (n=8 for each group; iWAT, *P*=0.4005; eWAT, *P*=0.000167; iBAT, *P*=0.676).

(c-d) ADCY3, ADCY4, ADCY6, p-HSL, HSL, p-PKA substrate, ATGL, IGFBP1 and GAPDH protein levels were measured by immunoblotting in the eWAT of *Wtap*^flox/flox^ and *Wtap-*HKO mice (n=4 for each group). ADCY3, ADCY4, ADCY6, p-HSL, HSL, p-PKA substrate, ATGL and IGFBP1 protein levels were quantified by Image J (n=4 for each group; ADCY3, *P*=0.00265; ADCY4, *P*=0.0193; ADCY6, *P*=0.0349; p-HSL/HSL, *P*=0.0057; p-PKA substrate/GAPDH, *P*=0.007; ATGL/GAPDH, *P*=0.0199; IGFBP1/GAPDH, *P*=0.00439). The samples were derived from the same experiment and the blots were processed in parallel.

(e) Serum IGFBP1 levels were measured by ELISA (n=8 for each group; *P*=0.0003).

n was the number of biologically independent mice. Data represent the mean ± SEM. Significance was determined by unpaired two-tailed Student's *t* test analysis. *, *p*< 0.05. **, *p*< 0.01. Source data are provided as a Source Data file.

**Supplementary Figure 6. IGFBP1 is associated with enhanced lipolysis in the eWAT of MCD-induced NASH.**

C57BL/6 mice were fed with a normal chow diet or an MCD diet for three weeks.

(a) Serum IGFBP1 protein levels were measured by ELISA (n=6 for each group; *P*<0.0001).

(b)IGFBP1, p-HSL, HSL, p-PKA substrate, ATGL and GAPDH protein levels in eWAT of normal chow and MCD feeding mice (n=4 for each group). The samples were derived from the same experiment and the blots were processed in parallel.

n was the number of biologically independent mice. Data represent the mean ± SEM. Significance was determined by unpaired two-tailed Student's *t* test analysis. **, *p*< 0.01. Source data are provided as a Source Data file.

**Supplementary Figure 7. *Ex vivo* neutralization of IGFBP1 in eWAT of *Wtap*-HKO mice decreases lipolysis**

eWAT was isolated from *Wtap*-HKO mice and cut into 30 mg pieces. Adipose tissue was incubated in RPMI1640 medium with different doses of anti-IGFBP1 antibody (0, 1, 5 μg) plus 1% BSA at 37℃ for 4 hours. Glycerol release was measured, and immunoblotting was performed.

(a) Glycerol release (n=3 for each group; 0 versus 5, *P*=0.0144).

(b) p-HSL, HSL, p-PKA substrate, ATGL and GAPDH protein levels were measured by immunoblotting. The samples were derived from the same experiment and the blots were processed in parallel.

n was the number of biologically independent tissue samples. These experiments were repeated for three times independently with similar results.

Data represent the mean ± SEM. Significance was determined by unpaired two-tailed Student's *t* test analysis. *, *p*< 0.05. **, *p*< 0.01. Source data are provided as a Source Data file.

**Supplementary Figure 8. IGFBP1 promotes lipolysis in** eWAT of ***Wtap*^flox/flox^, *Wtap*^flox/-^ Alb-Cre+(half deletion of *Wtap* in the liver), and *Wtap*^flox/flox^ Alb-Cre (*Wtap*-HKO) mice**

(a,b) eWAT was isolated from *Wtap*-HKO mice and cut into 30 mg pieces. Adipose tissue was incubated in RPMI1640 medium with different doses of IGFBP1 (0, 0.04, 0.1, 1, 2 μg) plus 1% BSA at 37℃ for 2 hours. Glycerol release (lipolysis) was measured (n=5 for each group). (b) is the basal (0) condition in (a) (n=5 for each group; *Wtap*^flox/flox^ versus *Wtap*-HKO *P*=0.001; *Wtap*^flox/-^ Alb-Cre+ versus *Wtap*-HKO *P*=0.0017). Their slope (k) values were analyzed. n was the number of biologically independent tissue samples. These experiments were repeated for three times independently with similar results.

(c) WTAP, IGFBP1 and Tubulin protein levels in the livers of *Wtap*^flox/flox^, *Wtap*^flox/-^ Alb-Cre+(half deletion of *Wtap* in the liver), and *Wtap*^flox/flox^ Alb-Cre (*Wtap*-HKO) mice were measured by immunoblotting. IGFBP1 and GAPDH protein levels in the eWAT of *Wtap*^flox/flox^, *Wtap*^flox/-^ Alb-Cre+(half deletion of *Wtap* in the liver), and *Wtap*^flox/flox^ Alb-Cre (*Wtap*-HKO) mice were measured by immunoblotting (n=3 for each group). The samples were derived from the same experiment and the blots were processed in parallel. n was the number of biologically independent mice. Data represent the mean ± SEM. Significance was determined by unpaired two-tailed Student's *t* test analysis. *, *p*< 0.05. **, *p*< 0.01. Source data are provided as a Source Data file.

**Supplementary Figure 9. IGFBP1 promotes lipolysis in** **primary adipocytes**

Fat stromal-vascular fraction (SVF) was isolated from 6-8 weeks old WT mice. For preadipocyte differentiation, cells grown to 100% confluence were exposed to induction in F12 medium containing 1 μM dexamethasone, 10 μg/mL insulin, 0.5μM isobutylmethylxanthine, and 10% FBS for 3-4 days. Cells were then differentiated in F12 medium containing containing10 μg/mL insulin and 10% FBS for 4-6 days. Fully differentiated primary adipocytes were used for lipolysis.

(a) Lipolysis (Glycerol release) was measured by glycerol reagent in primary adipocytes treated with different dose of IGFBP1 (n=3 for each group; o versus 1, *P*=0.0135).

(b) p-PKA substrate and GAPDH protein levels were measured by immunoblotting in primary adipocytes treated with different dose of IGFBP1. The samples were derived from the same experiment and the blots were processed in parallel.

n was the number of biologically cell samples. These experiments were repeated for three times independently.

Data represent the mean ± SEM. Significance was determined by unpaired two-tailed Student's *t* test analysis. *, *p*< 0.05. **, *p*< 0.01. Source data are provided as a Source Data file.

**Supplementary Figure 10. Hepatic deletion of *Wtap* decreases IGF1 but not GH expression.**

1. *GH* and *Wtap* mRNA levels were measured in pituitary gland of *Wtap*-HKO and *Wtap*^flox/flox^ mice at five weeks old (n=6 for each group; *GH*, *P*=0.4313; *Wtap*, *P*=0.3486).
2. *Igf1* mRNA levels were measured in livers of *Wtap*-HKO and *Wtap*^flox/flox^ mice at eight weeks old (n=8 for each group; *P*<0.0001).
3. Serum IGF1 levels were measured in *Wtap*-HKO and *Wtap*^flox/flox^ mice at eight weeks old (*Wtap*^flox/flox^, n=8; *Wtap*-HKO, n=9; *P*=0.0397).

n was the number of biologically independent mice. Data represent the mean ± SEM. Significance was determined by unpaired two-tailed Student's *t* test analysis. *, *p*< 0.05. **, *p*< 0.01. Source data are provided as a Source Data file.

**Supplementary Figure 11. WTAP regulates RNA m^6^A modification in the liver**

The m^6^A RNA immunoprecipitation sequencing(m^6^ARIP-seq) analysis was performed in the livers of 8-week-old *Wtap*^flox/flox^ and *Wtap-*HKO mice. (a) The enrichment of m^6^ARIP-seq peaks in the livers of 8-week-old *Wtap*^flox/flox^ and *Wtap-*HKO mice. (b) Consensus motif of m^6^A sites in livers of 8-week-old *Wtap*^flox/flox^. (c) The differential m^6^A peak enrichment related genes (*Wtap-*HKO VS *Wtap*^flox/flox^) including 2,314 down-regulated m^6^A peak related genes and 1,556 up-regulated m^6^A peak related genes were illustrated in a volcanoplot (|log2foldchange|>1 and pval<0.01). (d) GO analysis of the genes with downregulated m^6^A peaks.

**Supplementary Figure 12. Genes with downregulated m^6^A peaks are associated with signaling pathways.**

The m^6^A RNA immunoprecipitation sequencing(m^6^ARIP-seq) analysis was performed in the livers of 8-week-old *Wtap*^flox/flox^ and *Wtap-*HKO mice. KEGG analysis of the genes with downregulated m^6^A peaks.

**Supplementary Figure 13. Enrichment of open chromatin in the gene promoters and the ATAC-seq peaks near the TSS in the livers of *Wtap*^flox/flox^ and *Wtap-*HKO mice**  (a) Reads heatmap around TSS. (b) Reads profile around TSS.

**Supplementary Figure 14. Distribution of ChIP-seq peaks.**

**Supplementary Figure 15. KEGG analysis of downregulated genes in both ATAC-seq and RNA-seq (*Wtap-*HKO VS *Wtap*^flox/flox^).**

**Supplementary Figure 16. KEGG analysis of upregulated genes in both ATAC-seq and RNA-seq (*Wtap-*HKO VS *Wtap*^flox/flox^).**

**Supplementary Figure 17. WTAP is translocated from nuclei to cytosol in human NASH livers.**

Immunoblotting of WTAP protein levels in nuclei, cytosol and total tissue lysate from human NASH and normal liver tissues (n=5 for each group; Nuclei, *P*=0.0002; Cytosol, *P*=0.006976; Total tissue lysate, *P*=0.77959). The samples were derived from the same experiment and the blots were processed in parallel. n was the number of biologically liver samples. Data represent the mean ± SEM. Significance was determined by unpaired two-tailed Student's *t* test analysis. **, *p*< 0.01. Source data are provided as a Source Data file.

**Supplementary Figure 18. Quantification of serum palmitic acid concentration**

Source data are provided as a Source Data file.

Supplementary Table 1. Reagents and softwares used in this study.

| REAGENT or RESOURCE | SOURCE | IDENTIFIER |  |
| --- | --- | --- | --- |
| Antibodies |  |  | Dilution |
| METTL3 | CST | Cat#96391 | 1:2500 |
| WTAP | Proteintech | Cat#10200-1-AP | 1:2500 |
| IGFBP1 | ABclonal | Cat#A11672 | 1:3000 |
| F4/80 | CST | Cat#30325 | 1:200 |
| CD36 | Proteintech | Cat#18836-1-AP | 1:5000 |
| CCL2 | Proteintech | Cat#66272-1-Ig | 1:2500 |
| ATGL | Proteintech | Cat#55190-1-AP | 1:5000 |
| HSL | Abclonal | Cat#A15686 | 1:5000 |
| Phospho-HSL-S563 | Abclonal | Cat#AP0851 | 1:5000 |
| Phospho-(Ser/Thr)PKA Substrate | CST | Cat#9621 | 1:3000 |
| Rabbit IgG | Bioss | Cat#bs-0295p | Control antibody for neutralization |
| GAPDH | Proteintech | Cat#60004-1 | 1:5000 |
| β-Actin | Proteintech | Cat#60008-1-Ig | 1:5000 |
| Lamin B1 | Proteintech | Cat#12987-1-AP | 1:5000 |
| FLAG | Sigma | Cat#F1804 | 1:5000 |
| Cleaved-Caspase3 | CST | Cat#9661 | 1:2500 |
| HDAC1 | CST | Cat#5356 | 1:2500 |
| Acetyl-Histone H3 (Lys9) | Jingjie PTM BioLab | Cat#PTM-122 | 1:100 |
| Acetyl-Histone H3 (Lys27) | CST | Cat#4353 | 1:100 |
| CDK9 | Proteintech | Cat#11705-1-AP | 1:3000 |
| pCDK9 | CST | Cat#2549 | 1:3000 |
| Phosphoserine | Sigma | Cat#AB1603 | 1:1000 |
| ADCY6 | Proteintech | Cat#14616-1-AP | 1:3000 |
| ADCY4 | Bioss | Cat#bs-3921R | 1:3000 |
| ADCY3 | Bioss | Cat#bs-20272R | 1:3000 |
| MYC | Proteintech | Cat# 16286-1-AP | 1:5000 |
| αTubulin | Santa cruz | Cat#sc-5286 | 1:5000 |

| Chemicals | | |
| --- | --- | --- |
| TNFα | CUSABIO | Cat#CSB-AP002141HU |
| BAY-1143572 | Selleck | Cat#S8727 |
| Trichostatin A (TSA) | Selleck | Cat#S1045 |
| 3×Flag peptides | Sigma | Cat#F4799 |
| Protein A Agarose | Pierce | Cat#20333 |
| Anti-HA Magnetic Beads | Bimake | Cat#B26201 |
| Anti-FLAG M2 Magnetic Beads | Millipore | Cat#M8823 |
| Anti-MYC magnetic Beads | Bimake | Cat# B26301 |
| Glycerol Reagent | Sigma | Cat#F6428 |
| DAPI | Solarbio | Cat#C0060 |
| Palmitic acid | Sigma | Cat#P5585 |
| Blue Plus II Protein Marker | Transgen Biotech | Cat#L50824 |
| High Pure dNTPs | Transgen Biotech | Cat#20201025 |
| GoTaq Green Master Mix | Promega | Cat#M7122 |
| M-MLV reverse transcriptase | Promega | Cat#M1701 |
| Random Primers | Promega | Cat#C1181 |
| FBS | TRINTY TEK | Cat#01010102 |
| Collagenase Type 2 | Worthington | Cat#LS004176 |
| SYBR™ Green Mix | Roche | Cat#4913914001 |
| TriPure Isolation Reagent | Roche | Cat#94015120 |
| Recombinant DNase I | TaKaRa | Cat#2270A |
| Oil Red O | Sigma-Aldrich | Cat#O0625 |
| Direct Red 80 | Sigma-Aldrich | Cat#365548 |
| BODIPY Lipid Probes | Molecular Probes | Cat#MP 03792 |
| Recombinant Mouse IGFBP-1 Protein | R&D Systems | Cat#1588-B1-025 |
| MCD | Medicience | Cat#MD12052 |
| NASH diet | Changzhou SYSE Bio-Tec | Cat# D18402002 |

| Assay Kit | | |
| --- | --- | --- |
| TUNEL Assay Kit | Roche Applied Science | Cat#11684795910 |
| Alanine aminotransferase Assay Kit | Nanjing Jiancheng | Cat#C009-2-1 |
| Nonesterified Free fatty acids assay kit | Nanjing Jiancheng | Cat#A042-2-1 |
| Mouse IGFBP-1 ELISA Kit | BOSTER | Cat#EK0383 |
| Liver / Muscle glycogen assay kit | Nanjing Jiancheng | Cat#A043-1-1 |
| Mouse/Rat IGF1 ELISA Kit | Proteintech | Cat# KE10032 |
| Mouse MCP-1 ELISA Kit | Proteintech | Cat# KE10006 |
| Mouse IL-1 beta ELISA Kit | Proteintech | Cat# KE10003 |
| Mouse TNF-alpha ELISA Kit | Proteintech | Cat# KE10002 |
| cAMP ELISA kit | Cayman | Cat#581001 |

| Software | | |
| --- | --- | --- |
| Graphpad 6.02 | Graphpadsoftwave | Graphpad.com |
| Image J version 1.39f | National Institute of Health |  |

Supplementary Table 2. The clinical information and histologic features of subjects included in this study

|  |  | **Non-steatosis** |  | **NASH** |
| --- | --- | --- | --- | --- |
| All |  | 9 |  | 9 |
| Male Gender |  | 3(33%) |  | 8(89%) |
| Age (years) |  | 37.6±4.19 |  | 37.1±3.57 |
| BMI(kg/m2) |  | 21.3±0.41 |  | 24.4±1.42 |
| ALT(U/L) |  | 24.9±4.4 |  | 157±43.9 |
| AST(U/L) |  | 22.8±1.67 |  | 76.1±17.56 |
| Cholesterol (mM) |  | 3.93±0.3 |  | 4.63±0.3 |
| Triglycerides (mM) |  | 2.15±0.41 |  | 1.75±0.28 |
| HDL(mM) |  | 1.35±0.17 |  | 1.27±0.2 |
| LDL(mM) |  | 2.16±0.31 |  | 2.87±0.41 |
| FBG(mM) |  | 4.59±0.12 |  | 6.91±1.67 |
| Steatosis grade (1/2/3) |  | 0 |  | 1/5/3 |
| Lobular inflammation (1/2/3) |  | 0 |  | 4/3/2 |
| Ballooning (0/1/2) |  | 0 |  | 4/4/1 |
| Fibrosis (0/1) |  | 0 |  | 3/6 |

| Supplementary Table 3 Primers for qPCR | |  |
| --- | --- | --- |
| Genes | Forward | Reverse |
| Pparg | 5'-CCAGAGTCTGCTGATCTGCG-3' | 5'-GCCACCTCTTTGCTCTGATC-3' |
| Fasn | 5'-TTGACGGCTCACACACCTAC-3' | 5'-CGATCTTCCAGGCTCTTCAG-3' |
| Srebp1 | 5'-AACGTCACTTCCAGCTAGAC-3' | 5'-CCACTAAGGTGCCTACAGAGC-3' |
| Scd1 | 5'-AGGTGCCTCTTAGCCACTGA-3' | 5'-CCAGGAGTTTCTTGGGTTGA-3' |
| Cpt1α | 5'-CTGATGACGGCTATGGTGTTT-3' | 5'-GTGAGGCCAAACAAGGTGATA-3' |
| Ppara | 5'-CCTGAACATCGAGTGTCGAATA-3' | 5'-GGTCTTCTTCTGAATCTTGCAGCT-3' |
| Chrebp | 5'-CTGGGGACCTAAACAGGAGC-3' | 5'-GAAGCCACCCTATAGCTCCC-3' |
| mtGPAT1 | 5'-ACGCTGAGAGTGCCACATACT-3' | 5'-GAGAGATCGCTACAGCACCAC-3' |
| Dgat1 | 5'-CGTGGTATCCTGAATTGGTG-3' | 5'-GGCGCTTCTCAATCTGAAAT-3' |
| ApoB | 5'-CCAGAGTGTGGAGCTGAATGT-3' | 5'-TTGCTTTTTAGGGAGCCTAGC-3' |
| Mttp | 5'-CTCCACAGTGCAGTTCTCACA-3' | 5'-AGAGACATATCCCCTGCCTGT-3' |
| Mcad | 5'-ACCCTGTGGAGAAGCTGATG-3' | 5'-AGCAACAGTGCTTGGAGCTT-3' |
| Ccl2 | 5'-ACTGAAGCCAGCTCTCTCTTCCTC-3' | 5'-TTCCTTCTTGGGGTCAGCACAGAC-3' |
| Tnfα | 5'-CATCTTCTCAAAATTCGAGTGACAA-3' | 5'-TGGGAGTAGACAAGGTACAACCC-3' |
| Il1b | 5'-GCCTTGGGCCTCAAAGGAAAGAATC-3' | 5'-GGAAGACACGGATTCCATGGTGAAG-3' |
| 36B4 | 5'-AAGCGCGTCCTGGCATTGTCT-3' | 5'-CCGCAGGGGCAGCAGTGGT-3' |
| Il6 | 5'-AGCCAGAGTCCTTCAGA-3' | 5'-GGTCCTTAGCCACTCCT-3' |
| Ifng | 5'-GCTACACACTGCATCTTGGC-3' | 5'-CATGTCACCATCCTTTTGCCAG-3' |
| Cd36 | 5'-GGAGTGGTGATGTTTGTTGCT-3' | 5'-GCACACACCACCATTTCTTCT-3' |
| Fatp2 | 5'-CCAAAAGCGGCAACCATCAA-3' | 5'-AAGTAGCCCCAACCACGATG-3' |
| Fatp5 | 5'-TCCTTGGATTCCTTGGCTGC-3' | 5'-CTGGTTGCTCAGGGACGTTA-3' |
| Igfbp1 | 5'-GGGAGCCTGTGTACCAGAAC-3' | 5'-CTGATGGCGTTCCACAGGAT-3' |
| Tgfb1 | 5'-TTGCTTCAGCTCCACAGAGA-3' | 5'-TGGTTGTAGAGGGCAAGGAC-3' |
| Mmp9 | 5'-CGTCGTGATCCCCACTTACT-3' | 5'-AACACACAGGGTTTGCCTTC-3' |
| αSMA | 5'-GGAGAAGCCCAGCCAGTCGC-3' | 5'-AGCCGGCCTTACAGAGCCCA-3' |
| Collagen1a1 | 5'-TCACCTACAGCACCCTTGTG-3' | 5'-GGTGGAGGGAGTTTACACGA-3' |
| Cd14 | 5'-TACCGACCATGGAGCGTGTG-3' | 5'-CTGGACCAATCTGGCTTCGG-3' |
| Ccl3 | 5'-CCAAGTAGCCACATCGAGGG-3' | 5'-TGACCAACTGGGAGGGAGAT-3' |
| Ccl5 | 5'-CCACTTCTTCTCTGGGTTGG-3' | 5'-GTGCCCACGTCAAGGAGTAT-3' |
| Ccl22 | 5'-GGTGGCTCTCGTCCTTCTTG-3' | 5'-GTGACGGATGTAGTCCTGGC-3' |
| Ccr2 | 5'-GCCATCATAAAGGAGCCATACCT-3' | 5'-ATGCCGTGGATGAACTGAGG -3' |
| Cxcl2 | 5'-GAAGACCCTGCCAAGGGTTG-3' | 5'-AGGCAAACTTTTTGACCGCC-3' |
| Cxcl5 | 5'-TGCATTCCGCTTAGCTTTCT-3' | 5'-CAGAAGGAGGTCTGTCTGGA-3' |
| Cxcl10 | 5'-CCAAGTGCTGCCGTCATTTT-3' | 5'-CTCAACACGTGGGCAGGATA-3' |
| Cx3cl1 | 5'-GCAAGTTTGAGAAGCGGGTG-3' | 5'-CTTGGGAAGTCCCCATGGTC-3' |
| Csf1 | 5'-TGGCTTGGCTTGGGATGATT-3' | 5'-GTCTGTCCCCATGGTTTGGT-3' |
| Ccl2-ChIP | 5'-ACGAAGGAAACAGGGCAGAG-3' | 5'-AACGGGCCATGGAGAAACAT-3' |
| Cd36-ChIP | 5'-CACCTGAGCCAAATGAAATGAA-3' | 5'-CAGCAGAAAGAGGATTAAGGGT-3' |
| Igfbp1-ChIP | 5'-GCTGGATAAGCACGCAACAC-3' | 5'-CTGGGTGCTAGATCAGAGCG-3' |
| Actb-ChIP | 5'-AATAGCCTCCGCCCTTGTG-3' | 5'-CGTGACATCCACACCCAGA-3' |
| Adcy1 | 5'-GGCTGGAGGAGAAGCATTGT-3' | 5'-CGCCACCTATGGCAACACTA-3' |
| Adcy2 | 5'-TCAGGCATCATAGCCAACCG-3' | 5'-AGTCGGAAGGGTTGTCTCCT-3' |
| Adcy3 | 5'-GTTACACTGCCCCACCAAGT-3' | 5'-ACACCAGGACTTGGTCATTTGT-3' |
| Adcy4 | 5'-TGGAGCCTAGCTTTGCTGAG-3' | 5'-CTGTTGGCTCAGGCTGTAGT-3' |
| Adcy5 | 5'-GAAAATAATGCTGGCAGGAGCC-3' | 5'-TTTGCCTAAGCCGAGAGCAG-3' |
| Adcy6 | 5'-GCATCCTGTTTGCGGACATT-3' | 5'-ACAGTGATTCTCCCTCACCG-3' |
| Adcy7 | 5'-TGGGGACGTTCCCGAGAT-3' | 5'-GAGACATCTTCTTCCCTGCCG-3' |
| Adcy8 | 5'-CCGCAACGTCATCATCTTCG-3' | 5'-AGTACTCTGGGTAGGAGCAGA-3' |
| Adcy9 | 5'-GGTGTGTGTGGGCTTTTTCC-3' | 5'-GGAGTGGCCGTGAGAGTATG-3' |
| GH | 5'-ACTGCTCAGAGTCCTGTGGA-3' | 5'-GCTCGGAGCACAGCATTAGA-3' |
| Wtap | 5'-GCTTTGGAGGGAAAGTACAC-3' | 5'-CATCTCCTGCTCTTTGGTTG-3' |
| Igf1 | 5'-ATCTGCCTCTGTGACTTCTTGA-3' | 5'-TAGCCTGTGGGCTTGTTGAA-3' |

Supplementary Table 4. Multiple reaction monitoring (MRM) parameters of five free fatty acids.

| Analyte | Precursor ion  Q1(m/z) | Product ion  Q3(m/z) | Declustering potential (V) | Collision energy (V) |
| --- | --- | --- | --- | --- |
| Palmitic acid | 255.3 | 255.2 | -100 | -20 |
| Myristate | 227.3 | 227.1 | -108 | -20 |
| Stearate | 282.3 | 282.1 | -134 | -20 |
| Linoleate | 278.2 | 278.0 | -94 | -15 |
| Arachidonate | 303.2 | 259.4 | -125 | -20 |
