# Supplementary material for: Deficiency of WTAP in hepatocytes induces lipoatrophy and non-alcoholic steatohepatitis (NASH)
Source: Nat Commun. 2022 Aug 4;13:4549. doi: 10.1038/s41467-022-32163-w (PMC9352699; doi:10.1038/s41467-022-32163-w)
Supplement: Supplementary file 2 — Reporting Summary [file 41467_2022_32163_MOESM2_ESM.pdf]

nature portfolio

Corresponding author(s): Zheng Chen

Last updated by author(s): May 31, 2022

Reporting Summary

Nature Portfolio wishes to improve the reproducibility of the work that we publish. This form provides structure for consistency and transparency in reporting. For further information on Nature Portfolio policies, see our Editorial Policies and the Editorial Policy Checklist.

Statistics

For all statistical analyses, confirm that the following items are present in the figure legend, table legend, main text, or Methods section.

n/a	Confirmed	
☐	☒	The exact sample size (*n*) for each experimental group/condition, given as a discrete number and unit of measurement
☐	☒	A statement on whether measurements were taken from distinct samples or whether the same sample was measured repeatedly
☐	☒	The statistical test(s) used AND whether they are one- or two-sided<br>*Only common tests should be described solely by name; describe more complex techniques in the Methods section.*
☒	☐	A description of all covariates tested
☐	☒	A description of any assumptions or corrections, such as tests of normality and adjustment for multiple comparisons
☐	☒	A full description of the statistical parameters including central tendency (e.g. means) or other basic estimates (e.g. regression coefficient) AND variation (e.g. standard deviation) or associated estimates of uncertainty (e.g. confidence intervals)
☐	☒	For null hypothesis testing, the test statistic (e.g. *F*, *t*, *r*) with confidence intervals, effect sizes, degrees of freedom and *P* value noted<br>*Give P values as exact values whenever suitable.*
☒	☐	For Bayesian analysis, information on the choice of priors and Markov chain Monte Carlo settings
☒	☐	For hierarchical and complex designs, identification of the appropriate level for tests and full reporting of outcomes
☒	☐	Estimates of effect sizes (e.g. Cohen's *d*, Pearson's *r*), indicating how they were calculated

Our web collection on statistics for biologists contains articles on many of the points above.

Software and code

Policy information about availability of computer code

Data collection	RNA-seq, ChIP-seq, ATAC-seq and m6ARIP-seq analysis: TopHat version 2.0.12; HTSeq version 0.6.1; MACS2 Version 2.1.0; BWA Version 0.7.12; HOMER version 4.9.1; exomePeak R package version 2.16.0; fastp version 0.19.11; rMATS (version 3.2.5)<br>qPCR: LightCycler 480 Software 1.5.1.
Data analysis	GraphPad Prism 6.02; ImageJ version 1.39f

For manuscripts utilizing custom algorithms or software that are central to the research but not yet described in published literature, software must be made available to editors and reviewers. We strongly encourage code deposition in a community repository (e.g. GitHub). See the Nature Portfolio guidelines for submitting code & software for further information.

Data

Policy information about availability of data

All manuscripts must include a data availability statement. This statement should provide the following information, where applicable:

- Accession codes, unique identifiers, or web links for publicly available datasets
- A description of any restrictions on data availability
- For clinical datasets or third party data, please ensure that the statement adheres to our policy

ATAC-seq data that support the findings of this study have been deposited in GEO under accession code GSE168945 (https://www.ncbi.nlm.nih.gov/geo/query/acc.cgi?acc=GSE168945). RNA-seq data support the findings of this study have been deposited in GEO under accession code GSE168850 (https://www.ncbi.nlm.nih.gov/geo/query/acc.cgi?acc=GSE168850). m6ARIP-seq data that support the findings of this study have been deposited in GEO under accession code GSE192884(https://www.ncbi.nlm.nih.gov/geo/query/acc.cgi?acc=GSE192884). ChIP-seq data that support the findings of this study have been deposited in GEO under accession code GSE198023(https://www.ncbi.nlm.nih.gov/geo/query/acc.cgi?acc=GSE198023).

Field-specific reporting

Please select the one below that is the best fit for your research. If you are not sure, read the appropriate sections before making your selection.

☒ Life sciences ☐ Behavioural & social sciences ☐ Ecological, evolutionary & environmental sciences

For a reference copy of the document with all sections, see nature.com/documents/nr-reporting-summary-flat.pdf

Life sciences study design

All studies must disclose on these points even when the disclosure is negative.

Sample size	The sample sizes for this study were chosen based on generally expected variations of metabolic parameters and typical sample sizes for metabolic studies documented in literature(References: PMID: 24995979; 22315325; 22581287; 27131369; 27871061, 30613274 and 34893641). That allows to determine statistical differences in animal experiments and in vitro cell culture experiments. Sample numbers were well described in the Figure legends.
Data exclusions	No data were excluded from the analyses.
Replication	All the biological experiments were repeated, at least, three times and reproduced. Total RNA was extracted from the livers of Wtapflox/flox and Wtap-HKO mice at 8 weeks old, and three independent biological mice of each group were used for RNA-seq. For m6ARIP-seq, each sample (300 µg total RNA) was pooled from 5 mice for each group. Two independent biological replicates for each group were used for m6ARIP-seq. For ATAC-seq, each liver sample was pooled from three Wtapflox/flox and Wtap-HKO mice, respectively. Three independent biological replicates of each group were used for ATAC-seq. Four to ten independent samples were further validated by alternative approaches, such as RT-qPCR, ChIP-qPCR and luciferase assays. Western blotting data were confirmed by three to four independent samples.
Randomization	For animal experiments, age-matched mice with different genotypes were randomly divided into different experimental groups. For cell culture experiments, cells with different genotypes or treatments were randomly divided into different experimental groups.
Blinding	The H&E staining experiments were performed by the authors who were blinded to the experimental groups. ATAC-sequencing, RNA sequencing, m6ARIP-sequencing, library constructions, and RNA sequencing alignments were performed by technical staffs at Novogene who were blinded to the experimental groups. Blinding was not relevant to the other experiments in mice or cells because mice or cells had to be genotyped by PCR.

Reporting for specific materials, systems and methods

We require information from authors about some types of materials, experimental systems and methods used in many studies. Here, indicate whether each material, system or method listed is relevant to your study. If you are not sure if a list item applies to your research, read the appropriate section before selecting a response.

Materials & experimental systems

n/a	Involved in the study	
☐	☒	Antibodies
☐	☒	Eukaryotic cell lines
☒	☐	Palaeontology and archaeology
☐	☒	Animals and other organisms
☐	☒	Human research participants
☒	☐	Clinical data
☒	☐	Dual use research of concern

Methods

n/a	Involved in the study	
☐	☒	ChIP-seq
☒	☐	Flow cytometry
☒	☐	MRI-based neuroimaging

Antibodies

Antibodies used	Antibodies: WTAP Proteintech 10200-1-AP; IGFBP1 Abclonal A11672; F4/80 Cell Signaling Technology 30325; METTL3 Cell Signaling Technology 96391 D2160; FLAG Sigma F1804 M2; CD36 Proteintech 18836-1-AP; CCL2 Proteintech 66272-1-Ig 1B9F7 ; β-actin Proteintech 60008-1-Ig 7D2C10; Lamin B1 Proteintech 12987-1-AP; Cleaved Caspase3 Cell Signaling Technology 9661; HDAC1 Cell Signaling Technology 5356 10E2; Acetyl-Histone H3 (Lys9) Jingjie PTM BioLab PTM-112; Acetyl-Histone H3 (Lys27) Cell Signaling Technology 4353; Tubulin Santa cruz sc-5286 B-7; ATGL Proteintech 55190-1-AP; HSL Abclonal A15686; Phospho-HSL-S563 Abclonal AP0851; Phospho-(Ser/Thr) PKA Substrate CST 9621; GAPDH Proteintech 60004-1; Cleaved-Caspase3 CST 9661; CDK9 Proteintech Cat#11705-1-AP; pCDK9 CST Cat#2549; Phosphoserine Sigma Cat#AB1603; ADCY6 Proteintech Cat#14616-1-AP; ADCY4 Bioss Cat#bs-3921R; ADCY3 Bioss Cat#bs-20272R; MYC Proteintech 16286-1-AP; Rabbit IgG Bioss bs-0295p.
Validation	Most of the commercial antibodies have been verified and used in multiple previous publications as shown below.<br>WTAP ProteintechCat#10200-1-AP<br>https://www.ptgcn.com/products/WTAP-Antibody-10200-1-AP.htm

Species specificity: Human, mouse, rat, monkey
Applications: WB, IP
Publications: PMID: 34326314, 26190105, 34291881

F4/80 Cell Signaling Technology Cat#30325 D4C8V
https://www.cellsignal.cn/products/primary-antibodies/f4-80-d4c8v-xp-rabbit-mab/30325?site-search-type=Products&N=4294956287&Ntt=30325&fromPage=plp&_requestid=2615926
Species specificity: Mouse
Applications: WB, IP, IF
Publications: PMID: 32975669, 32900814, 33192521

METTL3 Cell Signaling Technology Cat#96391 D2I6O
https://www.cst-c.com.cn/products/primary-antibodies/mettl3-d2i6o-rabbit-mab/96391
Species specificity: Human, mouse, rat, monkey
Applications: WB, IP
Publications: PMID:31167133, 30212448, 32245957

FLAG Sigma Cat#F1804 M2
https://www.sigmaaldrich.cn/CN/zh/product/sigma/f1804?context=product
Species specificity: All
Applications: WB, IP, IHC, IF, ICC
Publications: PMID: 28053121,28328949,27172195

CD36 Proteintech Cat#18836-1-AP
https://www.ptgcn.com/products/CD36-Antibody-18836-1-AP.htm
Species specificity: Human, mouse, hamster
Applications: FC, IHC, WB,ELISA
Publications: PMID: 30233583,29664631,29025707

CCL2 Proteintech Cat#66272-1-Ig 1B9F7
https://www.ptgcn.com/products/Mcp1-Antibody-66272-1-Ig.htm
Species specificity: Mouse, Rat, Rabbit
Applications:IHC, WB,ELISA
Publications: PMID: 26978584,28943249,28129426

β-actin Proteintech Cat#60008-1-Ig 7D2C10
https://www.ptgcn.com/products/ACTB-Antibody-60008-1-Ig.htm
Species specificity: Human, mouse, rat, pig, plant, Zebrafish
Applications: FC, IF, IHC, WB,ELISA
Publications: PMID: 18667751,19699238,18596218
Lamin B1 Proteintech Cat#12987-1-AP
https://www.ptgcn.com/products/LMNB1-Antibody-12987-1-AP.htm
Species specificity: Human, mouse, rat
Applications: ChIP, IF, IHC, WB
Publications: PMID: 20132211,22745163,23146752

HDAC1 Cell Signaling Technology Cat#5356 10E2
https://www.cellsignal.cn/products/primary-antibodies/hdac1-10e2-mouse-mab/5356?site-search-type=Products&N=4294956287&Ntt=5356&fromPage=plp&_requestid=558238
Species specificity: Human, mouse, rat, monkey
Applications: WB, IP
Publications: PMID: 31924750,31885815,31849603

Acetyl-Histone H3 (Lys9) Jingjie PTM BioLab Cat#PTM-112
http://www.ptm-biolab.com.cn/productDetail.html?id=5656
Species specificity: Human, mouse, rat
Applications: WB, CHIP
Publications: PMID: 28429772, 25160476, 23909948

Acetyl-Histone H3 (Lys27) Cell Signaling Technology Cat#4353
https://www.cellsignal.cn/products/primary-antibodies/acetyl-histone-h3-lys27-antibody/4353?site-search-type=Products&N=4294956287&Ntt=4353&fromPage=plp&_requestid=558793
Species specificity: Human, mouse, rat, monkey
Applications: WB, IP, CHIP
Publications:PMID: 33397936, 32807777, 32025238

Tubulin Santa cruz Cat#sc-5286 B-7
https://www.scbt.com/zh/p/alpha-tubulin-antibody-b-7;jsessionid=Wa7VUc80f-fcWsAzlkec3W5PWv8SzKuYFJhw7AMBzcxfy9hKbmKf!-98738317

Species specificity: Human, mouse, rat, canine, bovine and porcine
Applications: WB, IP, IF, IHC, FCM, ELISA
Publications: PMID: 30755469; 31499479; 30810354

ATGL ProteintechCat#55190-1-AP
https://www.ptgcn.com/products/ATGL-Antibody-55190-1-AP.htm
Species specificity:Human, Mouse, Rat, Chicken, Pig, Sheep
Applications:IP, WB,ELISA
Publications:PMID: 29487348, 33529214, 29532859, 33605986

HSL AbclonalCat#A15686
https://abclonal.com.cn/catalog/A15686
Species specificity:Human, Mouse
Applications:WB
Publications: PMID: 31075194

Phospho-HSL-S563 AbclonalCat#AP0851
https://abclonal.com.cn/catalog/AP0851
Species specificity:Human, Mouse, Rat
Applications:WB
Publications:PMID: 31075194

Phospho-(Ser/Thr) PKA Substrate Cell Signaling TechnologyCat#9621
https://www.cellsignal.cn/products/primary-antibodies/phospho-ser-thr-pka-substrate-antibody/9621?site-search-type=Products&N=4294956287&Ntt=9621&fromPage=plp&_requestid=2694594
Species specificity:All
Applications:WB, IP, IHC
Publications:PMID: 33640452, 33934390, 34100382

GAPDH ProteintechCat#60004-1
https://www.ptgcn.com/products/GAPDH-Antibody-60004-1-Ig.htm
Species specificity:Human, Mouse, Rat, Yeast, Plant, Zebrafish
Applications:FC, IF, IP, WB, ELISA
Publications:PMID: 30293547, 30572886, 30506890

Cleaved Caspase3 Cell Signaling Technology Cat#9661
https://www.cellsignal.cn/products/primary-antibodies/cleaved-caspase-3-asp175-antibody/9661?_=1654040346473&Ntt=9661&tahead=true
Species specificity:Human, mouse, rat, monkey
Applications:WB, IP, IHC
Publications: PMID: 34031387,33931443

CDK9 Proteintech Cat#11705-1-AP
https://www.ptgcn.com/products/CDK9-Antibody-11705-1-AP.htm
Species specificity: Human
Applications: IHC, WB
Publications: PMID: 27315790,28474697

pCDK9 Cell Signaling Technology Cat#2549
https://www.cellsignal.cn/products/primary-antibodies/phospho-cdk9-thr186-antibody/2549?site-search-type=Products&N=4294956287&Ntt=2549&fromPage=plp&_requestid=556627
Species specificity: Human, mouse, rat, monkey
Applications: WB
Publications: PMID: 31848275,31101827,29792310

Phosphoserine Sigma Cat#AB1603
https://www.sigmaaldrich.cn/CN/zh/product/mm/ab1603?context=product
Species specificity: All
Applications: ELISA, WB, IP, IHC
Publications: PMID: 11181844,12960006,15514089

ADCY6 Proteintech Cat#14616-1-AP
https://www.ptgcn.com/products/ADCY6-Antibody-14616-1-AP.htm
Species specificity:Human, Mouse, Rat
Applications:WB, IP, IHC
Publications:PMID: 31383768,31918924

ADCY4 Bioss Cat#bs-3921R;
http://www.bioss.com.cn/prolook_03.asp?id=AF08169606003382&pro37=1

Species specificity:Human, Mouse, Rat, Dog, Pig, Cow, Horse, Rabbit, Sheep
Applications:WB, ELISA
Publications: used in this paper

ADCY3 Bioss Cat#bs-20272R
http://www.bioss.com.cn/prolook_03.asp?id=AF08169606025014&pro37=1
Species specificity:Human, Mouse, Rat,Pig, Cow, Horse, Sheep
Applications:WB, ELISA
Publications: used in this paper

MYC ProteintechCat#16286-1-AP
https://www.ptgcn.com/products/MYC-tag-Antibody-16286-1-AP.htm
Species specificity: MYC-Tag antibody
Applications:IF, IP, WB,ELISA
Publications: PMID: 25906440,28069035,28598244

Rabbit IgG BiossCat#bs-0295p
http://www.bioss.com.cn/prolook_03.asp?id=AF08169606005429&pro37=7
Applications:Isotype Control
Publications: PMID: 34133792, 35132073, 30685705

IGFBP1Abclonal Cat#A11672
https://abclonal.com.cn/catalog/A11672
Species specificity: Human, mouse
Applications: WB
Publications: used in this paper

Eukaryotic cell lines

Policy information about cell lines

Cell line source(s)	HEK293T ATCC CRL3216
Authentication	The cell line has not been authenticated recently.
Mycoplasma contamination	All cell lines were tested negative for mycoplasma contamination.
Commonly misidentified lines (See ICLAC register)	There is no ICLAC line used in this study.

Animals and other organisms

Policy information about studies involving animals; ARRIVE guidelines recommended for reporting animal research

Laboratory animals	Male mice were used for experiments. Age is between 5 to 20 weeks. The age of mice for each experiment was shown in the relevant figure legends. Both WTAPflox/flox and Alb-Cre mice were in C57BL/6J background. Animals were kept under controlled light (12hour light and 12hour dark cycle), temperature (24 ± 2°C) and humidity (50% ± 10%) conditions. The strain and housing temperature of mice were described in 'Methods' .
Wild animals	This study did not involve wild animals.
Field-collected samples	This study did not involve samples collected from the field.
Ethics oversight	Animal experiments were carried out in strict accordance with the Guide for the Care and Use of Laboratory Animals published by the US National Institutes of Health and approved by the Institutional Animal Care and Use Committee or Animal Experimental Ethics Committee of Harbin Institute of Technology (HIT/IACUC). The permit number was IACUC-2018004.

Note that full information on the approval of the study protocol must also be provided in the manuscript.

Human research participants

Policy information about studies involving human research participants

Population characteristics

The clinical information and histologic features of subjects included in this study was shown in Supplementary Table 1.
The information was also shown below.
 Non-steatosis NASH
All 9 9
Male Gender 3(33%) 8(89%)
Age (years) 37.6±4.19 37.1±3.57
BMI(kg/m2) 21.3±0.41 24.4±1.42

	ALT(U/L) 24.9±4.4 157±43.9<br>AST(U/L) 22.8±1.67 76.1±17.56<br>Cholesterol (mM) 3.93±0.3 4.63±0.3<br>Triglycerides (mM) 2.15±0.41 1.75±0.28<br>HDL(mM) 1.35±0.17 1.27±0.2<br>LDL(mM) 2.16±0.31 2.87±0.41<br>FBG(mM) 4.59±0.12 6.91±1.67<br>Steatosis grade (1/2/3) 0 1/5/3<br>Lobular inflammation (1/2/3) 0 4/3/2<br>Ballooning (0/1/2) 0 4/4/1<br>Fibrosis (0/1) 0 3/6
Recruitment	All the participants in this study were enrolled between March 2018-June 2021 at the 3rd affiliated hospital of Sun Yat-sen university. No expect biases, including self-selection bias was expected from patient samples. The investigation conforms to the principles that are outlined in the Declaration of Helsinki regarding the use of human tissues.
Ethics oversight	The present study were approved by the Research Ethics Committee of the Third Affiliated Hospital of Sun Yat-sen University

Note that full information on the approval of the study protocol must also be provided in the manuscript.

ChIP-seq

Data deposition

☒ Confirm that both raw and final processed data have been deposited in a public database such as GEO.

☒ Confirm that you have deposited or provided access to graph files (e.g. BED files) for the called peaks.

Data access links<br>*May remain private before publication.*	ChIP-seq data that support the findings of this study have been deposited in GEO under accession code GSE198023(https://www.ncbi.nlm.nih.gov/geo/query/acc.cgi?acc=GSE198023). The secure token for review purpose is "edurwqsafpedryn".
Files in database submission	GSM5935997 WTAP-IP<br>GSM5935998 WTAP-Input<br>GSE198023_Wtap_peaks.xlsx
Genome browser session<br>(e.g. UCSC)	We used GRCm38.p6 as genome reference.

Methodology

Replicates	ChIP-sequencing was performed once for one replicates but four independent samples were analyzed and further validated by ChIP-qPCR .
Sequencing depth	Paired-ends; For total number of reads, WTAP-IP:24168697; WTAP-Input:22718375; For unique_mapped reads, WTAP-IP:20634444; WTAP-Input:20484973; Length of reads: 272
Antibodies	We used Anti-FLAG M2 Magnetic Beads (M8823, Millipore) for ChIP-seq experiment.
Peak calling parameters	After mapping reads to the reference genome, we used the MACS2 (version 2.1.0) peak calling<br>software to identify regions of IP enrichment over background. The parameter is "-q 0.05; --call-summits; --nomodel". After peak calling, the distribution of chromosome distribution, peak width, fold enrichment,<br>significant level and peak summit number per peak were all displayed.
Data quality	We used the MACS2 (version 2.1.0) peak calling software to identify regions of IP enrichment over background with a parameter "-q 0.05; --call-summits; --nomodel". We detected 27225 peaks.
Software	fastp (version 0.19.11); BWA (v 0.7.12); MACS2 (version 2.1.0); HOMER (version 4.9.1)
