# Supplementary material for: Deficiency of WTAP in hepatocytes induces lipoatrophy and non-alcoholic steatohepatitis (NASH)
Source: Nat Commun. 2022 Aug 4;13:4549. doi: 10.1038/s41467-022-32163-w (PMC9352699; doi:10.1038/s41467-022-32163-w)
Supplement: Supplementary file 3 — Description of Additional Supplementary Files [file 41467_2022_32163_MOESM3_ESM.pdf]

Title: Supplementary Data 1.
RNA-seq analysis shows that 344 transcripts display significant alternative splicing changes in the livers of *Wtap*-HKO mice.

Alternative splicing was analyzed by rMATS software (version 3.2.5). False Discovery Rate (FDR) <0.05 was considered statistically significant.

Title: Supplementary Data 2.
m^6ARIP-seq analysis shows that transcripts with significant m^6A peaks come from 9,020 genes in the livers of *Wtap*$^{flox/flox}$ mice.

Title: Supplementary Data 3.
ATAC-seq data analysis identified 15,903 upregulated peak-related genes and 16,042 downregulated peak-related genes in the livers of *Wtap*-HKO mice.

Title: Supplementary Data 4.
ChIP-seq analysis identifies 15,266 genes with peaks in Flag-WATP-overexpressing hepatocytes.

Title: Supplementary Data 5.
Combined analysis of ATAC-seq and RNA-seq data show that 953 genes are downregulated in these two datasets, whereas 1,532 genes are upregulated in the livers of *Wtap*-HKO mice.
